# Radmis, a Novel Mitotic Spindle Protein that Functions in Cell Division of Neural Progenitors

**DOI:** 10.1371/journal.pone.0079895

**Published:** 2013-11-08

**Authors:** Takahito Yumoto, Kazuhiko Nakadate, Yuki Nakamura, Yoshinobu Sugitani, Reiko Sugitani-Yoshida, Shuichi Ueda, Shin-ichi Sakakibara

**Affiliations:** 1 Laboratory for Molecular Neurobiology, Graduate School of Human Sciences, Waseda University, Tokorozawa, Saitama, Japan; 2 Department of Basic Biology, Educational and Research Center for Pharmacy, Meiji Pharmaceutical University, Kiyose-shi, Tokyo, Japan; 3 Department of Cell Biology, Cancer Institute, The Japanese Foundation for Cancer Research, Koto-ku, Tokyo, Japan; 4 RIKEN Brain Science Institute, Wako, Saitama, Japan; 5 Department of Molecular Immunology and Inflammation, Research Institute, National Center for Global Health and Medicine, Shinjuku-ku, Tokyo, Japan; 6 Department of Histology and Neurobiology, Graduate School of Medicine, Dokkyo Medical University, Mibu, Tochigi, Japan; 7 Institute of Applied Brain Sciences, Waseda University, Tokorozawa, Saitama, Japan; University of Queensland, Australia

## Abstract

Developmental dynamics of neural stem/progenitor cells (NSPCs) are crucial for embryonic and adult neurogenesis, but its regulatory factors are not fully understood. By differential subtractive screening with NSPCs versus their differentiated progenies, we identified the radmis (*radial fiber and mitotic spindle*)/ckap2l gene, a novel microtubule-associated protein (MAP) enriched in NSPCs. Radmis is a putative substrate for the E3-ubiquitin ligase, anaphase promoting complex/cyclosome (APC/C), and is degraded via the KEN box. Radmis was highly expressed in regions of active neurogenesis throughout life, and its distribution was dynamically regulated during NSPC division. In embryonic and perinatal brains, radmis localized to bipolar mitotic spindles and radial fibers (basal processes) of dividing NSPCs. As central nervous system development proceeded, radmis expression was lost in most brain regions, except for several neurogenic regions. In adult brain, radmis expression persisted in the mitotic spindles of both slowly-dividing stem cells and rapid amplifying progenitors. Overexpression of radmis *in vitro* induced hyper-stabilization of microtubules, severe defects in mitotic spindle formation, and mitotic arrest. *In vivo* gain-of-function using *in utero* electroporation revealed that radmis directed a reduction in NSPC proliferation and a concomitant increase in cell cycle exit, causing a reduction in the Tbr2-positive basal progenitor population and shrinkage of the embryonic subventricular zone. Besides, radmis loss-of-function by shRNAs induced the multipolar mitotic spindle structure, accompanied with the catastrophe of chromosome segregation including the long chromosome bridge between two separating daughter nuclei. These findings uncover the indispensable role of radmis in mitotic spindle formation and cell-cycle progression of NSPCs.

## Introduction

During mammalian central nervous system (CNS) development, neural stem/neural progenitor cells (NSPCs) generate neural and glial lineages by mitotic cell division. At the early embryonic stage, neuroepithelial cells spanning the neural tube serve as primary NSPCs. As the neuroepithelium thickens, neuroepithelial cells differentiate into radial glial cells (apical progenitors), and shift their mode of proliferation from symmetric to asymmetric cell division [1-3].. Similar to neuroepithelial cells, these cells undergo cell division at the ventricular zone (VZ), and display a defined apico-basal polarity with a radially oriented fiber (radial process) extending from the VZ to the pial surface of the cortical wall [4]. Meanwhile, another type of neural progenitor cell, called intermediate progenitors or basal progenitors, originate from asymmetric divisions of radial glial cells. Basal progenitors delaminate from the VZ to form a second proliferative layer, the subventricular zone (SVZ), during the late embryonic stage. In the perinatal stage, radial glial cells differentiate into ependymal cells that face the ventricular system [5]. The SVZ persists into adulthood in a considerably reduced form. In the adult rodent SVZ, slowly dividing glial fibrillary acidic protein (GFAP)-positive cells are thought to be neural stem cells (NSCs; type-B cells) that give rise to rapidly proliferating progenitors (type-C cells) [2,6]. 

Persistent maintenance of NSPC lineages throughout life might indicate shared molecular machinery among NSPCs [7]. Substantial changes of the microtubule network in NSPCs may play the principal role in this machinery. Microtubules assemble into the highly organized mitotic spindle at the entry of mitosis of NSPCs [8], in addition to their involvement in the architecture of radial cell processes. During neurogenesis, programmed timing and the frequency of spindle formation of NSPCs determines the total number of neurons and brain size [9]. Furthermore, it is now clear that positioning of the mitotic spindle into the cleavage plane determines daughter cell fate by symmetric/asymmetric segregation of cell fate determining factors such as m-Numb [10]. As a group of proteins that directly modulate the stability and function of microtubules, there is increasing interest in the role of microtubule-associated proteins (MAPs) during neural development [11]. Growing evidence suggests that several MAPs, including DCLK [12] and ASPM [13,14], play vital roles not only in NSPC division, but also in the neuronal fate determination of their progeny during neurogenesis. 

In the present study, we report a novel mitotic spindle protein named radmis that is highly expressed in NSPCs. Radmis protein emerges at the mitotic-phase of cell cycle through the post-translational regulation. The constitutive expression or knockdown of radmis perturbs the cell division of NSPCs with the aberrant mitotic spindles, and results in the abnormal cell-fate of their progenies. Tightly controlled expression of radmis is essential for the maintenance of dividing NSPCs during neurogenesis. 

## Materials and Methods

### Ethics statement

This study was carried out in strict accordance with the recommendations in the Guide for the Care and Use of Laboratory Animals of the National Institutes of Health. The protocol was approved by the Committee on the Ethics of Animal Experiments of the Waseda University. All surgery was performed under sodium pentobarbital anesthesia, and all efforts were made to minimize suffering.

### Animals and tissue preparation

ICR mice, used for the preparation of tissue protein extracts, RNA, or tissue sections, were obtained from Takasugi Experimental Animals Supply (Saitama, Japan) or SLC (Shizuoka, Japan). The date of conception was established by the presence of a vaginal plug and recorded as embryonic day zero (E0.5) and the day of birth was designated as P0.

### NSPC culture

Primary cortical NSPC culture was prepared from cerebral cortices of E11.5 embryos or SVZ of 8 weeks-old adult male mice. Mechanically dissociated cells of telencephalons or SVZ were seeded onto fibronectin and poly-L-ornithine (Sigma-Aldrich Japan, Tokyo, Japan)-coated dishes, and cultured for 5 days in DMEM/F-12 (1:1) supplemented with 15 µg/ml insulin (Life technologies, Carlsbad, CA), 25 µg/ml transferrin (Life technologies), 20 nM progesterone (Sigma-Aldrich), 30 nM sodium selenite (Sigma-Aldrich), 60 nM putrescine (Sigma-Aldrich), 20 ng/ml FGF2 and 10 ng/ml EGF (Merck Millipore) at 37°C in a humidified atmosphere of 5% CO_2_. NSPCs culture were then replated at 1×10^5^ per 10-cm dish, and further expanded for 4 days in the presence of FGF2 and EGF before induction of differentiation. Before differentiation, all stem cells divide and express nestin, an intermediate filament characteristic of neuroepithelial precursors [15]. Sister culture of NSPCs was induced to differentiate by withdrawal of FGF2 and EGF, and addition of 10% fetal bovine serum for 3 days. Neurosphere cultures were prepared as described previously [16].

### Suppression subtractive hybridization (SSH)

SSH libraries were constructed using the PCR-Select™ cDNA Subtraction Kit and PCR-Select™ Differential Screening Kit (Clontech Takara Bio, Shiga, Japan), according to the manufacturer’s suggested conditions [17]. Poly (A)^+^ RNA (2.0 µg) from the stem cell culture and differentiated sister culture were purified using oligotex-dT30 Super (Takara Bio, Shiga, Japan), and used for synthesis of tester (St) and driver (Dif) cDNA, respectively. After second strand cDNA synthesis, samples were digested with *Rsa*I to generate shorter, blunt-ended, double-stranded cDNA fragments, necessary for adaptor ligation. Both forward and reverse subtraction experiments were performed, which selected for genes that were enriched in the stem/progenitor population compared with the differentiating population. *Rsa*I-digested tester cDNA was ligated with either adaptor 1 or adaptor 2R, denatured and then hybridized with excess driver cDNA pool. Fresh denatured driver DNA was added, and a second hybridization was performed. The samples were amplified by PCR; the primary PCR was 27 cycles with the Primer 1 (5’-CTAATACGACTCACTATAGGGC-3’), and the secondary was 12 cycles with Nested Primer 1 (5’-TCGAGCGGCCGCCCGGGCAGGT-3’) and Nested Primer 2R (5’-AGCGTGGTCGCGGCCGAGGT-3’), according to the manufacturer’s instruction. RT-PCR analysis of the SSH products showed that the level of the house-keeping gene GAPDH decreased more than 1,000 fold in subtracted cDNA pool when compared with unsubtracted cDNA (data not shown). After secondary PCR amplification, PCR products from the forward subtraction were cloned into the pT7-Blue vector (Merck Millipore) and cloned into *E. coli* DH5α to generate the subtractive library. To perform the differential screening and analysis of cloned SSH cDNAs, cDNA insert fragments were amplified directly from each bacterial colonies using Nested Primers 1 and 2R, and spotted onto Hybond-N nylon membranes (GE Healthcare Japan, Tokyo, Japan) in duplicate and probed with ^32^P-labeled probes made from either the forward subtracted or reverse subtracted cDNA pool using the High Prime DNA labeling kit (Roche Applied Science, Indianapolis, IN). 120 clones demonstrating significant differential expression in the stem/progenitor pool were identified and sequenced to prioritize further characterization. Sequence homology searches and alignments were performed with BLAST algorithms on the NCBI server.

### Screening of genes expressed in NSPCs

Among clones of most differentially expressed in subtracted libraries, northern blot was conducted on 20 clones that encode novel genes or genes with unknown function. Total RNA from the neural stem cell culture and from the differentiated sister culture for 7 div in the presence of 10% serum were blotted and probed with the radiolabeled insert fragment of each SSH cDNAs as describe bellow. *In situ* hybridization screening was then performed onto tissue sections from developing mouse at a variety of stages ranging from early embryonic day 13.5 to adult, using clones identified by northern blot screening as being differentially expressed.

### Northern and western blot analysis

Northern and western blot analysis was performed as described previously [18,19]. For northern blot, total RNA (20 µg) from each of the mouse brain or cultured cells was isolated using Trizol (Life technologies) according to the manufacturer’s instructions. Each SSH cDNA insert fragment or a 750-bp 3’-UTR fragment of the mouse *radmis* cDNA were used to prepare ^32^P-labelled probe. The integrity and equal loading of the RNA samples was verified by reprobing each blot with a radiolabeled *g3pdh* cDNA fragment.

### *In situ* hybridization of mRNA

*In situ* hybridization of mRNA was performed on 6-µm paraffin sections or 20-µm frozen sections from E13.5 embryos, P6 pups and 8-weeks old adult brains using digoxigenin-labeled riboprobes, according to the manufacturer’s instructions (Roche). Each SSH cDNA insert fragment was subcloned into pBluescript II KS vector (Agilent Technologies, Santa Clara CA) and used to prepare the riboprobes, with DIG RNA Labeling (Roche). *In vitro* transcription was carried out with the T7 or T3 RNA polymerases (Promega, Madison, WI).

### *radmis* cDNA

Mouse *radmis* cDNAs were isolated by RT-PCR using RNA isolated from E12.5 and adult brains. Mouse MGC (mammalian gene collection) verified cDNA (IRAT) clone BC053443 (IMAGE: 6333921) contained the full-length *mouse radmis* (*m-radmis*) cDNA sequence were purchased from Open Biosystems (Huntsville, AL). Human expressed sequence tags (EST) clone AL832036 contained the full-length *human radmis* (*h-radmis*) cDNA sequence were obtained from ImaGenes GmbH (Berlin, Germany).

### Mammalian expression vectors

*radmis* cDNAs were subcloned into the vector pEGFP-C2 (Clontech Takara Bio) to express the radmis protein fused with enhanced green fluorescent protein (EGFP) on its N-terminus (pEGFP-*m-radmis* and pEGFP-*h-radmis*), and used for the transfection experiments in mammalian cells. For *in utero* electroporation, full-length *m-radmis* cDNA and EGFP-*m-radmis* cDNA were placed into the expression vector pCAGGS (kindly provided by Dr. Jun-ichi Miyazaki, Osaka University, Japan), in which the chicken β-actin promoter coupled to the CMV enhancer drives the transgene expression [20]. In order to monitor the efficiency of *in utero* electroporation, pCAGGS-EGFP and pCAGGS-DsRed-Express were constructed from the green fluorescent protein vector pEGFP and red fluorescent protein vector pDsRed-Express (Clontech Takara Bio), respectively, and co-electroporated in some experiments. KEN-box mutation of mouse radmis (amino acid residues 183 to 185) was generated using the QuikChange II XL Site-Directed Mutagenesis Kit (Agilent Technologies) according to the manufacturer’s instructions. The following primers were used for mutagenesis: K183A reverse (5'-gcagggcctggggcaaggctgctgcgtttgtttcatctggaaagccatccaca-3'), and K183A forward (5'-tgtggatggctttccagatgaaacaaacgcagcagccttgccccaggccctgc-3').

### Cell culture transfection

Neuro2a (N2a), HEK293, and NIH3T3-13C7 cells were grown in Dulbecco’s modified Eagle’s medium (Life technologies) with 10% (v/v) fetal bovine serum. Cells were grown on coverslips pre-coated with 100 µg/ml poly-L-lysine (Sigma-Aldrich). DNA transfection was performed using Lipofectamine LTX (Life technologies) as instructed by the manufacturers. At 42 h post-transfection, cells were fixed with 4% PFA for 20 min at 4°C, and permeabilized in 0.05% Triton X-100 in PBS for 10 min, and then subjected to the immunostaining analysis. The cell cycle was monitored by the morphology of chromosomes stained with both anti-phosphoH3 antibody and Hoechst dye (Sigma-Aldrich).

### Production of polyclonal antibody to radmis

The 593-bp *Bam*HI-*Eco*RI fragment corresponding to the carboxy terminal 197 amino acids of mouse radmis protein was isolated by RT-PCR of the RNAs of E12 embryonic brains, and subcloned in-frame into the pGEX-2T vector (GE Healthcare) to make a glutathione S-transferase (GST) fusion protein. About 200 μg of GST-Radmis fusin protein was affinity purified by the Glutathion-Sepharose 4B resin (GE Healthcare) and used to immunize New Zealand White rabbits. For the purification of antibody, same 593-bp *radmis* cDNA fragment was subcloned into pET32a vector (Merck Millipore) to generate the Thioredoxin (Trx) and His-tagged fusion protein. Trx-His-tagged radmis protein purified with the Ni Sepharose 6 Fast Flow (GE Healthcare) was coupled covalently to with the HiTrap NHS-activated HP column as described by the supplier (GE Healthcare). Ten milliliter of the filtered (0.45 µm) whole antisera was incubated with 5 ml of the affinity resin pre-equilibrated with binding buffer (0.5 M NaCl, 20 mM Tris-HCl, pH7.5) overnight at 4°C. The resin was then washed with 250 ml of binding buffer, followed by 20 ml of 0.15M NaCl, eluted with 4 ml of 100 mM glycine-HCl, pH 2.5 at 4°C, and immediately neutralized with 0.4 ml of 1 M Tris-HCl (pH 8.5).

### Primary antibodies

The following primary antibodies were used: radmis (affinity-purified rabbit polyclonal antibody, 1:10000 for immunostaining, 1:100000 for immunobloting); nestin (mouse monoclonal IgG, clone Rat 401, Developmental Studies Hybridoma Bank of Iowa University, 1:250), βIII-tubulin (mouse monoclonal IgG_2a_, clone TuJ1, Covance Japan, Tokyo, Japan, 1:500), NeuN (mouse monoclonal IgG_2a_, Merck Millipore, 1:500), glial fibrillary acidic protein (GFAP) (mouse monoclonal IgG_1_, clone G-A-5, Sigma-Aldrich, 1:1000), Dlx-2 (anti guinea pig IgG, 1:2000 kindly gifted from Dr. Kazuaki Yoshikawa, Osaka Univ.)[21], α-tubulin (mouse monoclonal, clone B-5-1-2, Sigma-Aldrich, 1:2000), γ-tubulin (mouse monoclonal IgG, clone GTU-88, Sigma-Aldrich, 1:4000), phospho-Histone 3 (Ser10) (mouse monoclonal IgG_1_, clone 6G3, Cell Signaling Technology, 1:500), pericentrin (monoclonal IgG, clone 28144, Abcam, UK, 1:1000). EGFP (rabbit polyclonal, Life Technologies, 1:1000), EGFP (chick IgY, AvesLab, Oregon, 1:1000), Tbr2 (chick IgY, Merck Millipore, 1:1000), Pax6 (rabbit polyclonal, MBL, Nagoya, Japan, 1:1000), anti-bromodeoxyuridine (BrdU) (sheep polyclonal, Abcam, 1:1500), Ki-67 (rabbit monoclonal clone SP6, Lab Vision, CA, 1:1000).

### Immunostaining

Immunohistochemical analysis was performed as described previously [18,22]. Immunolabeling with a single primary antibody was performed on paraffin sections (6 µm thickness) or free floating frozen sections (30 µm thickness) using the avidin-biotin-peroxidase technique (Vectastain ABC Elite kit, Vector Laboratories, Burlingame, CA), according to the manufacturer’s instructions. Immunostained sections were counterstained with methyl green or hematoxylin. For control sections, the anti-radmis antibody was omitted or replaced with normal rabbit serum. The specificity of the anti-radmis antibody was examined by its preadsorption with a His-tagged radmis recombinant protein (100 pmole/ml), expressed in *E. coli*, before its use for immunostaining tissue sections. For the indirect dual immunofluorescence staining, Alexa Fluor 488-, Alexa Fluor 568-, or Alexa Fluor 647-conjugated secondary antibodies used at 1:2000 dilution (Life technologies). For primary antibodies generated from chicken, DyLight 488, or DyLight 549 -conjugated anti-chick IgY was used at 1:1000 dilution (Jackson Immuno Research Lab, West Grove, PA). Hoechst 33342 (Sigma-Aldrich), TOPRO-3 (Life technologies), or propidium iodide (Sigma-Aldrich) were used for nuclear staining. Specimens were examined under a fluorescence microscope Axio Observer equipped with ApoTome module (Carl Zeiss Microscopy). Optical sections were viewed using a scanning laser confocal imaging system Fluoview FV500 (Olympus, Tokyo, Japan) or TCS4D (Leica Microsystems, Bensheim, Germany). For BrdU/Ki-67 double staining, frozen sections were pretreated with 2 N HCl for 30 min at 42°C to denature the DNA, followed by 0.1 M sodium borate buffer, pH 8.5, for 10 min. After three washes with PBS, indirect double-immunolabeling was performed as described above.

### Electron microscopy

Adult male ICR mice were transcardially perfused with fixative containing 4% PFA, 0.2% picric acid, and 0.01% glutaraldehyde in 0.1 M phosphate buffer (PB), pH 7.4. After the brains were rinsed with 0.1 M PB, sections were cut on a VT1000S microtome (Leica Microsystems) at a thickness of 50 µm, and cryoprotected in a solution containing 30% sucrose in 0.1M PB. The sections were freeze-thawed and incubated in 1% H_2_O_2_ solution for 2 h. After washing, the sections were incubated in a blocking solution containing 1% normal goat serum in PBS for 2 h, followed by the incubation with rabbit anti-radmis antibody (diluted 1:20,000) in PBS overnight. After washing, the sections were incubated with biotinylated goat anti-rabbit IgG antibody, then reacted with the avidin-biotin peroxidase complex (Vector Laboratories) and reacted with DAB/H_2_O_2_ in Tris-HCl buffer, pH 7.6. After treatment with OsO_4_, sections were embedded in Epon-812 resin (TAAB, Switzerland) after dehydration with a graded ethanol series. Ultrathin sections were prepared (Ultramicrotome MT-XL, RMC, Tucson, AZ) and not stained with uranyl acetate or lead citrate to omit false-positive staining. These specimens were examined with a JEM-1011 electron microscope (JEOL, Tokyo, Japan).

### TUNEL staining

For apoptosis in embryonic brains, TUNEL assays were performed using the In Situ Cell Death Detection Kit, TMR-red (Roche), according to the manufacturer’s instruction. The percentages of TUNEL positive cells were calculated among the EGFP-positive electroporated cells.

### *In utero* electroporation

Timed pregnant E14.5 mice were anesthetized with sodium pentobarbital. Embryos were exposed in the uterus, and 5µg/µl DNA solution with 0.01% Fast Green dye (Sigma-Aldrich) was injected into the lateral ventricle through the uterus wall via the pulled glass capillaries, followed by the electroporation. Following expression constructs driven by CAG promoter were electroporated; pCAGGS-EGFP-*radmis*, pCAGGS-EGFP-*radmis-mutant*, or control plasmid pCAGGS-EGFP, pCAGGS-DsRed-Express. Electric pulses were generated by Super Electroporator NEPA21 (Nepagene, Chiba, Japan) and applied to the cerebral wall with five pulses of 36 V for 50 msec with an interval of 950 msec. The voltage was discharged across platinum oval electrode (CUY650P5, Nepagene) placed on the uterine wall across the head of the embryo. An anionic electrode was placed on the lateral cortex to ensure that DNA incorporated into the VZ/SVZ. For BrdU labeling experiments, pregnant dams were injected daily with BrdU solution (100 mg/kg body weight i.p.) from E15.5 to E17.5. Embryos were perfused at E17.5 with 4% PFA through cardiac perfusion. Frozen sections were immunostained with anti-EGFP antibody in conjunction with the appropriate primary antibodies.

### shRNAmir expression vectors

We purchased pGIPZ lentiviral vector library, which encode the microRNA-adapted shRNA (shRNAmir) targeting mouse radmis (ckap2l), from Thermo Scientific Open Biosystems. Among seven clones (V2LMM-93654, V2LMM-93655, V2LMM-197689, V3LMM-485979, V3LMM-485981, V3LMM-485983, V3LMM-485984), V3LMM-485983 and V3LMM-485984 were identified as the shRNA that can efficiently knockdown the endogenous radmis in cultured cells, and designated as shRNA#1, and shRNA#3, respectively. Targeting sequences for shRNA#1 and shRNA#3 are TGGTCGTGTAGAATCTGCA, and TTGTTAGCTCTGTCTTTCA, respectively. Both of them are located at the ORF region of radmis gene. Non-silencing scrambled shRNA (# RHS4346) was also purchased from Open Biosystems. To further generate the CAG-promoter based expression vector of shRNAmir, MluI-XhoI fragment (300 bp) of each clone was subcloned into the NotI site of pCAG-EGFP, in which radmis or scrambled shRNAmir is expressed as an IRES-regulated polysistronic gene together with EGFP and puromycin resistance gene.

### Data analysis

For the *in vitro* transfection, at least three independent transfection experiments were performed using the cultured cells grown on the cover slips. 30-40 random fields from each coverslip imaged with a 40× objective were photographed, and EGFP-positive cells were counted (10-16 cover slips were used for each transfection condition). Data were pooled for each experimental condition, tested for significance by Student’s *t*-test or unpaired *t*-test, and presented as mean ± SEM (%). The number of cells used for statistical analysis was indicated in Figure legends. For the quantitative analysis of the *in utero* electroporated embryos, 4-15 animals were analyzed for each group. Optical images of 5-7 sections per embryo, at least 60 µm apart, and at the same neuroanatomical level in each group, were captured by confocal microscopy equipped with a 20 - 40× objective. Data were pooled for each embryo, tested for significance by Student’s *t*-test or unpaired *t*-test and expressed as the mean ± SEM (%). For the quantification of radmis-positive cells in the adult SVZ, the serial coronal sections (free floating frozen sections at 30 µm thickness) were prepared from at least 3 male animals. Immunostained sections were assessed by conducting cell counts on every 6th section from the level of anterior commissure through the fimbria. To distinguish the SVZ region, the nuclear staining was simultaneously performed. Images were obtained using the confocal microscopy with a 40× objective.

### Accession number

The nucleotide sequence data reported in this paper have been deposited with the GSDB, DDBJ, EMBL, and NCBI under accession no. AB455263 for mouse *radmis* cDNA.

## Results

### Identification of the *radmis* gene

NSPCs can be isolated from the developing and adult mouse brain, and are preferentially enriched in fibroblast growth factor 2 (FGF2)- and epidermal growth factor (EGF)-containing culture medium [23]. Prior to differentiation, NSCs divide and express nestin, an intermediate filament characteristic of neuroepithelial precursors [15]. Under differentiating conditions of growth factor withdrawal and the addition of 10 % fetal bovine serum, the proportion of multipotent progenitors within cultures dramatically decreases, and these cells differentiate efficiently into neurons, astrocytes, and oligodendrocytes. To isolate genes that are expressed by developing NSPCs, we used the suppression PCR-subtractive hybridization technique using RNAs from undifferentiated NSPCs (tester) and differentiated cells (driver). We used adherent FGF2- and EGF-expanded NSPCs derived from the telencephalon of E11.5 embryos or the adult mouse SVZ as the tester cDNA pool, and each sister culture under a differentiating condition for 3 days for the driver cDNA pool (see Materials and Methods). Among 2000 candidate cDNAs that were enriched in subtracted libraries, 120 clones were verified to be differentially expressed in the cDNA pool of NSPCs. All of these clones were sequenced, and their mRNA expressions in NSPCs were examined independently by northern blot and *in situ* hybridization using embryonic and postnatal brains. As a result, we isolated six clones (MG46, MB61, ME55, ME85, SD35, and SE90) that encode novel proteins or proteins with unknown function, which showed restricted mRNA expression in the germinal zones such as the embryonic VZ and adult SVZ. We focused on the characterization of SE90 in the present study. 

**Figure 1:**
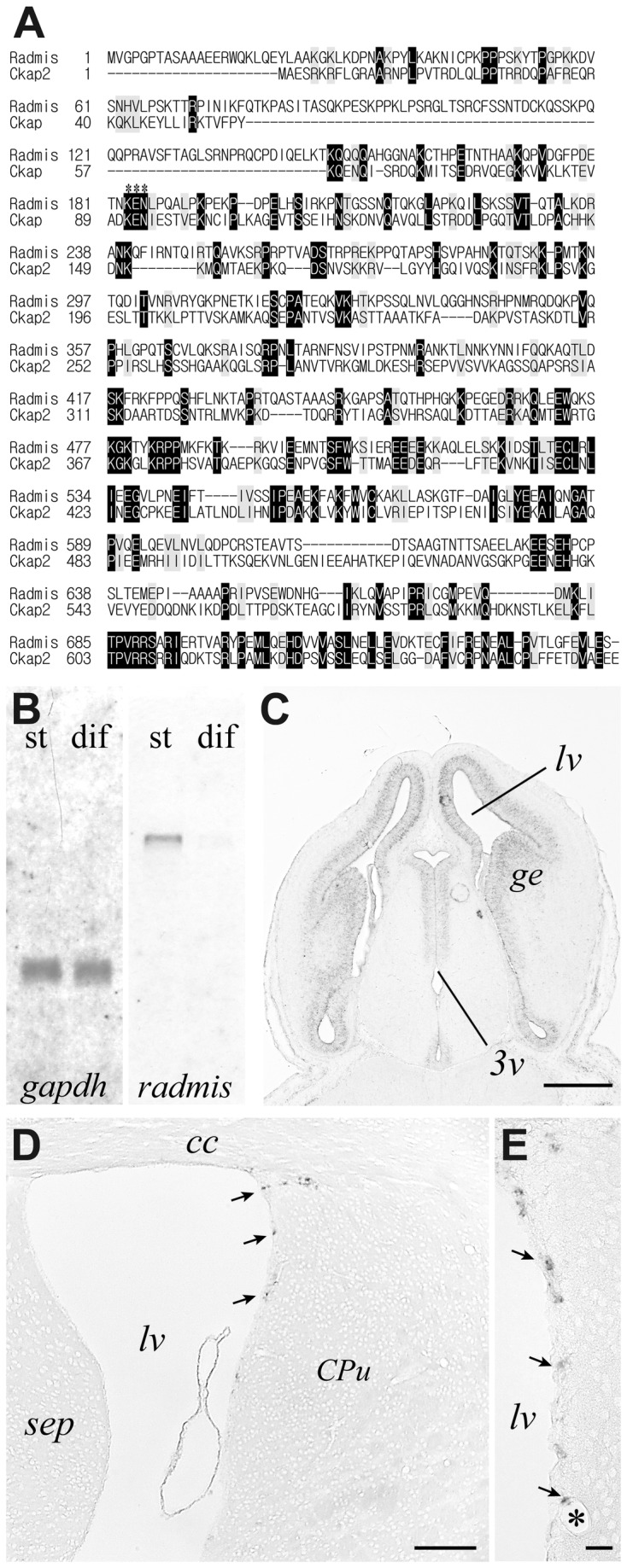
Cloning of radmis gene. (**A**) Primary structure of mouse radmis, and the alignment with mouse ckap2 is shown. Identical amino acids are highlighted, and similar amino acid residues are shaded in gray. Gaps in the alignment are indicated by dashes. Asterisks denote the KEN box sequence. (**B**) Northern blot analysis. *St*, NSCs expanded in monolayer culture; *dif*, differentiating cells. Equal loading of RNAs was verified by probing with *gapdh* (*right panel*). (**C**-**E**) *In* *situ* hybridization for the *radmis* mRNA. Horizontal section of E 13.5 embryonic brain showing *radmis* expression in the VZ surrounding lateral ventricles (lv) and the 3^rd^ ventricle (3v) (**C**). Coronal section of adult forebrain (**D**). Magnified view of the adult SVZ (**E**) showing each *radmis*-positive cell (*arrows*) sparsely distributed in the lateral wall SVZ of the lateral ventricle. Bars: *C*, 180 μm; *D*, 100 μm; *E*, 20 μm. *lv*, lateral ventricle; *3v*, third ventricle; *ge*, ganglionic eminence; *cc*, corpus callosum; *str*, striatum; *asterisk*, blood vessel.

A BLAST homology search against the GenBank database showed that the SE90 gene was identical to the cytoskeleton associated protein 2-like (ckap2l ) gene that has been deposited in DNA databases as one of the many genes induced during growth factor-mediated muscle cell survival [24]. At present, there is no experimental evidence regarding the ckap2l gene expression profile, or a putative function of the ckap2l gene. The protein product of the ckap2l gene has been predicted to show similarity with that of TMAP/ckap2 [25]. However, as shown in Figure 1, our sequence analysis using the clustalW2 program revealed that the SE90/ckap2l protein shares only 16% amino acid identity with that of TMAP/ckap2. Thus, we designated the SE90/ckap2l gene as “*radmis*” because of its preferential distribution in the radial fiber and mitotic spindle in NSPCs *in vivo* as described below. The open reading frame of *radmis* mRNA encodes a 745 amino acid protein with a predicted molecular mass of 84 kDa. Although radmis is an evolutionarily conserved protein found in various vertebrate species (chicken, dog, mouse, rat, cow, monkey, and human), we could not find any functional domains in the radmis protein. Northern blot hybridization using a mouse radmis cDNA probe revealed that a radmis transcript of 3.2 kb was expressed abundantly in the stem cell population, and was rapidly down-regulated during differentiation (Figure 1B). *In situ* hybridization experiments further confirmed high level expression of *radmis* mRNA in the embryonic VZ (Figure 1C). In adult mice, *radmis* mRNA expression was absent in most CNS areas except for the SVZ, where NSPCs reside. A few *radmis*-expressing cells were detected in the SVZ surrounding the lateral ventricles (Figure 1D, E). To determine whether *radmis* mRNA is expressed in a tissue-specific manner, we performed northern blotting of RNA isolated from various adult mouse tissues by probing with a P^32^-labeled cDNA. However, the radmis transcript was undetectable in all tested tissues including brain, lung, liver, kidney, heart, and skeletal muscles (data not shown), possibly due to the low expression level.

### Radmis is expressed by NSPCs

**Figure 2:**
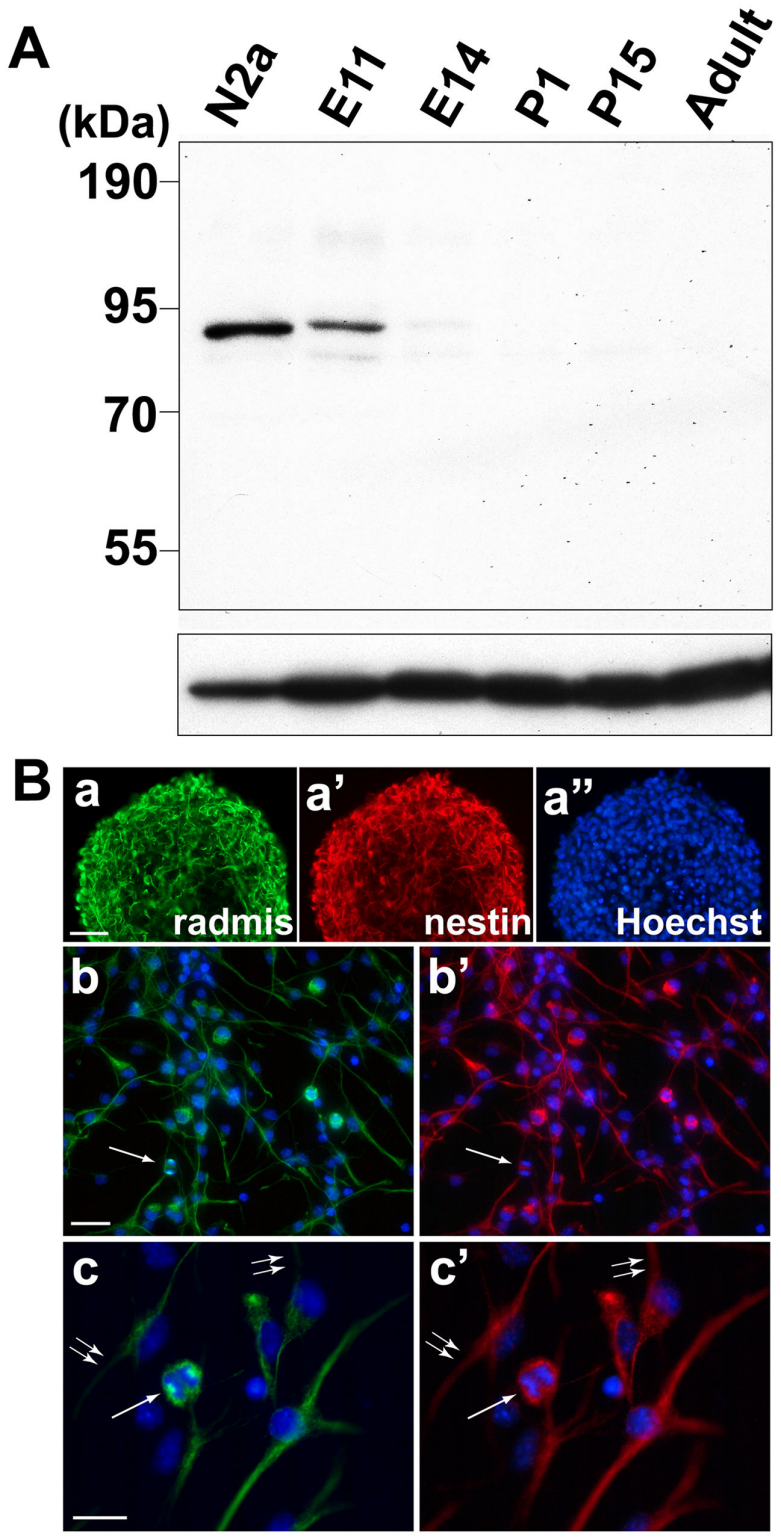
Protein expression of radmis in the developing brain and NSPCs. (**A**) Developmental time course of radmis expression in the brain. The level of radmis was determined by immunoblot using radmis antibody. Radmis expression was detected as a major band of 85 kDa at E11. Lane N2a shows a total cell lysate from the mouse neuroblastoma cell line N2a. The blot was reprobed with an anti-α-tubulin antibody (*bottom panel*) to confirm the equal loading. (**B**) Radmis protein expression in cultured NSPCs. (*a*) Double immunostaining of a neurosphere derived from an E14.5 embryonic telencephalon showing coexpression of radmis (*a, green*) and nestin (*a’, red*). Nuclei were counterstained with Hoechst (*a*”*, blue*). (*b*, *c*) Cultured NSPCs expanding as a monolayer and immunostained for radmis (*b, c, green*) and nestin (*b’, c’, red*). Hoechst (*blue*). (*c*) Higher magnification view of (*b*). Radmis is detected in the cell body during mitosis (*arrow*) and in the long fine processes of NSPCs that are nestin-positive. Note that the protein distributions of radmis and nestin in cellular processes did not completely overlap with each other (*double arrows*). Scale bars: *a*, 50 μm; *b*, 50 μm; *c*, 25 μm.

To examine the intracellular distribution of radmis protein, a rabbit polyclonal antibody was raised against a bacterially expressed recombinant mouse radmis. As shown in Figure 2A, the radmis antiserum recognized single polypeptides of approximately 84 kDa in immunoblots, which is the expected size of radmis. The immunoblot revealed that the content of radmis protein drastically decreased during brain development (Figure 2A). Expression of endogenous radmis protein was only observed at E11.5, when active expansion of NSPCs occurs. Thereafter, its expression level was rapidly decreased until E14.5, and undetectable in adult brain.

To assess the distribution of radmis protein in an NSPC population, we performed immunostaining experiments with cultured NSPCs that were isolated from E14.5 mouse cortices and maintained in a defined medium containing FGF2 and EGF. In the presence of EGF and FGF2, dissociated embryonic neural cells proliferate and form floating neurospheres [16,26]. Most of the cells in a neurosphere are clonally derived from a single NSPC, and are thought to possess the characteristics of stem cells, namely self-renewal and multipotent differentiation into neurons and glia [26]. As shown in Figure 2B, radmis was expressed in the cells of neurospheres derived from E14.5 telencephalon. These neurospheres were also uniformly co-immunolabeled with an anti-nestin antibody. NSPCs cultured as a monolayer frequently exhibit unipolar or bipolar processes (Figure 2B), resulting in a morphology resembling that of neuroepithelial cells in the embryonic VZ. Double immunostaining of radmis and nestin in monolayer NSPC cultures showed that radmis was expressed throughout the cytoplasm and cellular processes. These results confirmed the abundant expression of radmis in a population of NSPCs *in vitro*. In cultured dividing NSPCs, radmis expression was found to be closely associated with mitotic spindles (Figure 2Bc). In addition, radmis and nestin were coexpressed in the extending long processes of interphase NSPCs, although their distributions did not completely overlap with each other. Nestin is uniformly distributed in the relatively thick cell processes, while radmis immunoreactivity seems to be more restricted in the delicate and thinner structure within the process (Figure 2Bc, *double arrows*). Considering that nestin is a type VI cytoplasmic intermediate filament protein, radmis might be related to the cytoskeletal component that is different from the intermediate filament in NSPCs. 

### Expression of radmis during embryonic CNS development

**Figure 3:**
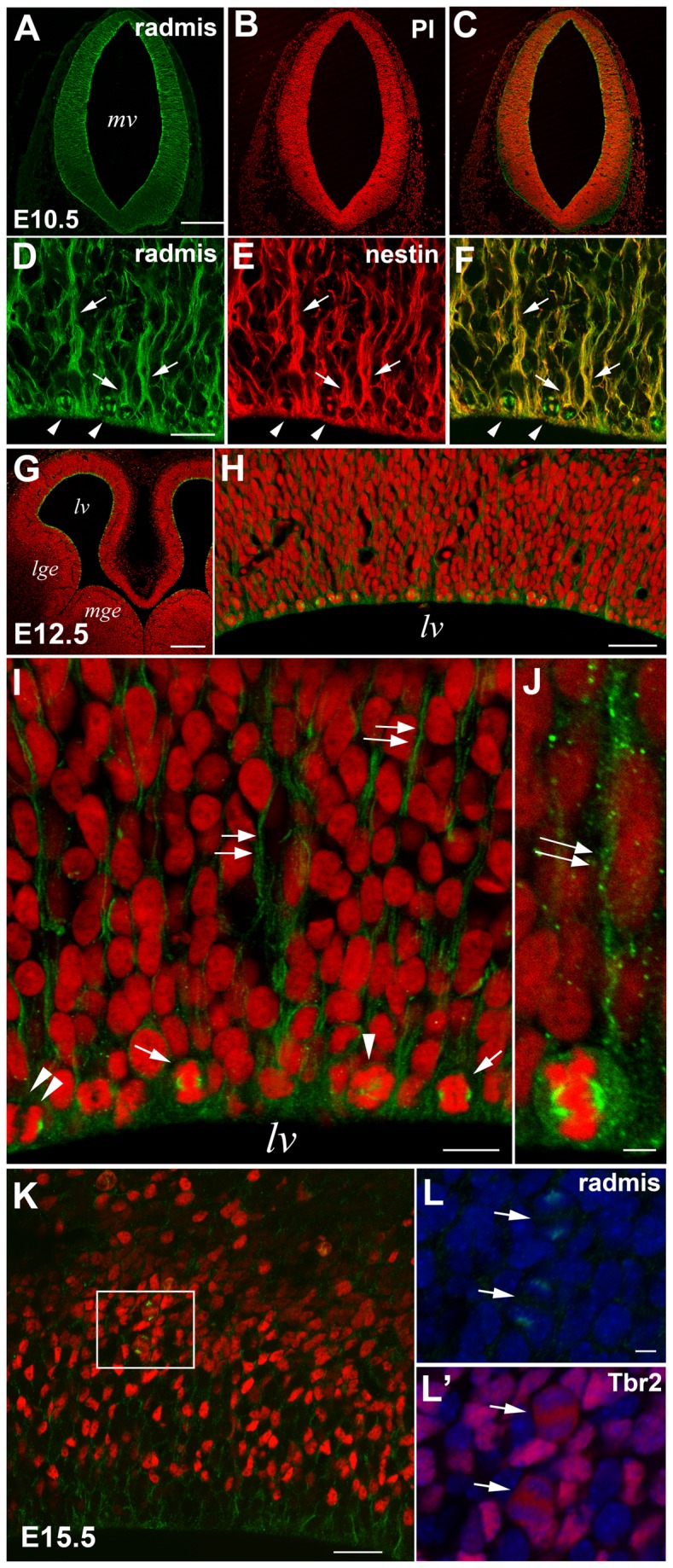
Radmis expression during embryonic CNS development. (**A**–**C**) E10.5 neural tube of the metencephalon immunostained for radmis (**A** *, green*) and counterstained with propidium iodide (PI) (***B**, red*), showing uniformly distributed radmis in neuroepithelial cells throughout the neural tube. Note the significantly lower immunoreactivity of radmis in the connective tissue outside the neural tube. (**D**–**F**) Higher magnification view of the ventricular region of an E10.5 neural tube double-stained for radmis (***D**, green*) and nestin (***E**, red*). Radmis is expressed in the mitotic spindles (*arrowheads*) and nestin-positive radial fibers (*arrows*) of neuroepithelial cells. (**G**–**J**) E12.5 forebrain immunostained for radmis (*green*) and PI stained (*red*). (**H**) Cerebral neocortex at E12.5. (**I**) Magnified view of the ventricular surface of (H) showing accumulation of radmis in the mitotic spindles (*arrowhead*, prometaphase; *arrows*, metaphase; *double* *arrowhead*, anaphase) of dividing NSPCs located at the ventricular surface, and their radial fibers (*double arrows*). (**J**) A dividing NSC at metaphase showing robust distribution of radmis in the mitotic spindle and its extending radial fiber (*double arrows*). (**K**–**L**) E15.5 neocortex immunostained for radmis (*green*) and Tbr2 (*red*). (**L**, **L**’) Higher magnification of the boxed area in K. *Arrows* depicted the radmis immunoreactivity (**L**) in mitotic spindles of Tbr2 (**L**’)-positive dividing cells within the SVZ. Chromosome staining (*blue*) indicated that these two dividing cells were in anaphase. Scale bars: *A*–*C*, 250 μm; *D*–*E*, 20 μm; *G*, 200 μm; *H*, 31 μm; *I*, 12 μm; *J*, 3 μm; *K*, 250 μm; *L*, 5 μm. *lv*, lateral ventricle; *mge*, medial ganglionic eminence; *lge*, lateral ganglionic eminence.

To address whether radmis expression is restricted to NSPCs *in vivo*, we immunostained embryonic brain sections with an anti-radmis antibody. Embryonic NSPCs, also called neuroepithelial cells or radial glia, span the entire cortical wall. Previous studies demonstrated that radial glia undergo symmetric cell divisions to expand the number of progenitor cells, or asymmetric cell divisions to generate postmitotic neurons that eventually migrate to the cerebral cortex [1,4,10]. At E10.5, when the neural tube closes, radmis expression was mainly observed in the neuroepithelial cells of the neural tube (Figure 3A). Microscopic inspection using a higher magnification revealed that radmis expression was frequently associated with the mitotic spindles of proliferating neuroepithelial cells and their radially aligned processes, which are immunopositive for nestin, and extend their processes from the VZ to the outer part of the neural tube (Figure 3D–F). As CNS development proceeds, many differentiated neurons occupy peripheral locations of the cortex, but a substantial number of NSPCs are sustained in the VZ as radial glia [1,3,27]. Accordingly, immunolocalization of radmis was confined to the dividing NSPCs (radial glia) lining the surface of the ventricular wall at E12.5 (Figure 3G, H). Radmis was intensely expressed in the mitotic spindles of dividing NSPCs, as well as their radial fibers extending from the cell body (*double arrows* in Figure 3I, J). The expression of radmis in mitotic spindles was also observed in the Tbr2-positive basal progenitors in SVZ [28] during the late embryonic stage (Figure 3K, L). Such an intracellular distribution of radmis protein was comparable with that observed in NSPCs *in vitro* (Figure 2B). These data suggest that radmis predominantly functions in the mitotic spindles and cellular processes of NSPCs.

### Radmis expression in the early postnatal CNS

**Figure 4:**
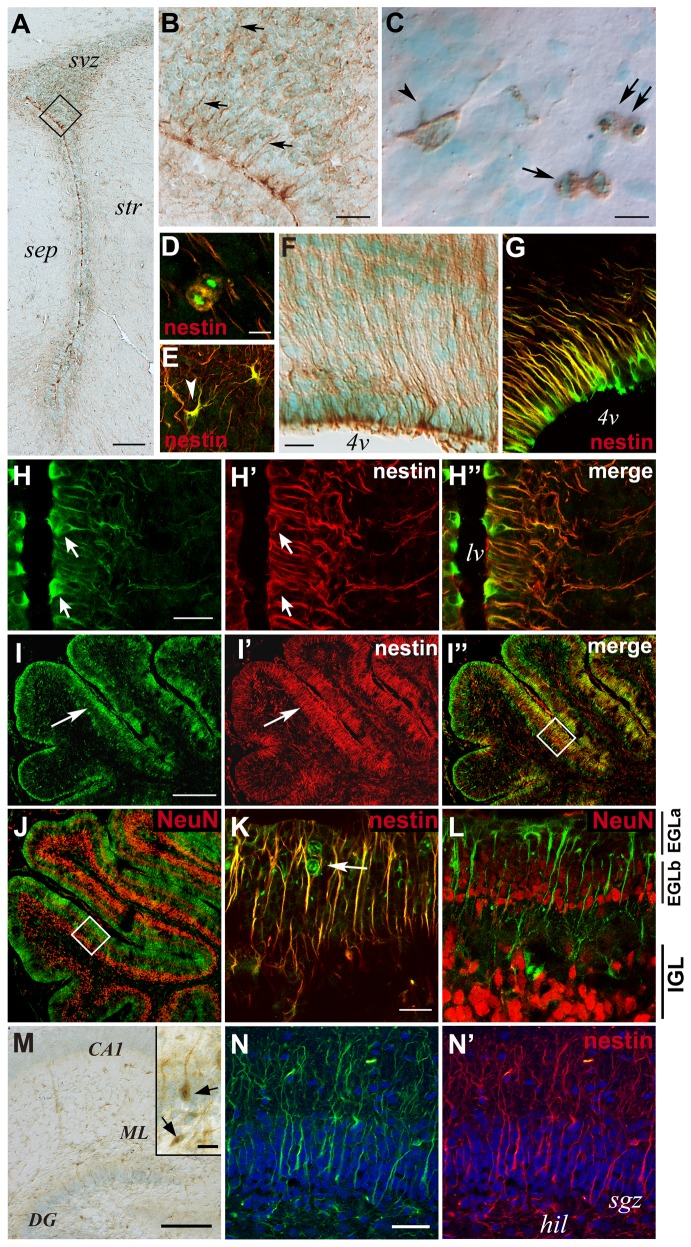
Radmis expression in postnatal brain. (**A**–**C**) Coronal sections of P6 forebrain were immunostained for radmis (*brown*), followed by counterstaining with methyl green. (**A**) Low-power view of the SVZ region. (**B**) Magnification of the boxed area in A. *Arrows* indicate radmis immunoreactivity in fine processes extending from the lateral ventricle. (**C**) Individual radmis-positive cells within the SVZ. Radmis is detected in cells with short processes (*arrowhead*), or during mitosis (*arrows*). (**D**, **E**) Double immunostaining of SVZ cells for radmis (*green*) and nestin (*red*). (**F**, **G**) Pontine area surrounding the 4^th^ ventricle and immunostained for radmis (**F**). (**G**) Double-immunostaining for radmis (*green*) and nestin (*red*). (**H**) Lateral and medial wall surrounding the lateral ventricle, stained for radmis (*green*) and nestin (*red*). *Arrows* indicate radmis expression in nestin-positive immature ependymal cells and their processes. (**I**–**L**) Radmis expression in the postnatal cerebellum. Double-immunofluorescence labeling of sagittal sections through the P6 cerebellum, with antibodies against radmis (*green*) and nestin or NeuN (*red*). (**I**) Co-immunostaining with nestin. Radmis is highly expressed in densely packed cells in the EGL (**I**, *arrowheads*), which also express nestin (**I**”). (**J**) Double-labeling showing non-overlapping localization of radmis and NeuN. NeuN expression is prominent in the IGL and is faint in the EGL. (**K**, **L**) Higher magnification of the boxed area in I and J, respectively. The EGL is toward the top of the panels. Radmis is highly expressed in mitotic cells (*arrow*) residing in the outer zone of the EGL (EGLa), whereas NeuN is expressed in differentiating post-mitotic neurons in the inner zone of the EGL (EGLb), in addition to the granule neurons in the IGL. Note that radmis is also expressed in the nestin-positive radial fibers of developing Bergmann glia, coursing through the EGL. (**M**, **N**) Radmis expression in the hippocampus at P7. (M) Low-power view showing the radmis expression (*brown*) in the CA and DG regions. Inset shows the magnified view of the radmis-positive cells in DG. *Arrows* indicate mitotic cells. (**N**, **N**’) Double immunostaining of the P7 DG with nestin. Radmis is expressed in nestin-positive cells bearing the radially aligned process through the developing granular layer. Scale bars: *A*, 100 μm; *B*, 30 μm; *C*–*E*, 5 μm; *F, G*, 10 μm; *H*, 12 μm; *I, J*, 140 μm; *K, L*, 20 μm; *M*, 100 μm; *M* *inset*, 20 μm; *N*, 30 μm. *sep*, septum; *str*, striatum; *4v*, 4^th^ ventricle; *IGL*, internal granule layer; *EGL*, external granule cell layer; *CA1*, pyramidal layer of CA1 region; *DG*, dentate gyrus; *ML*, molecular layer of DG; *hil*, hillus; *sgz*, subgranular zone.

**Figure 5:**
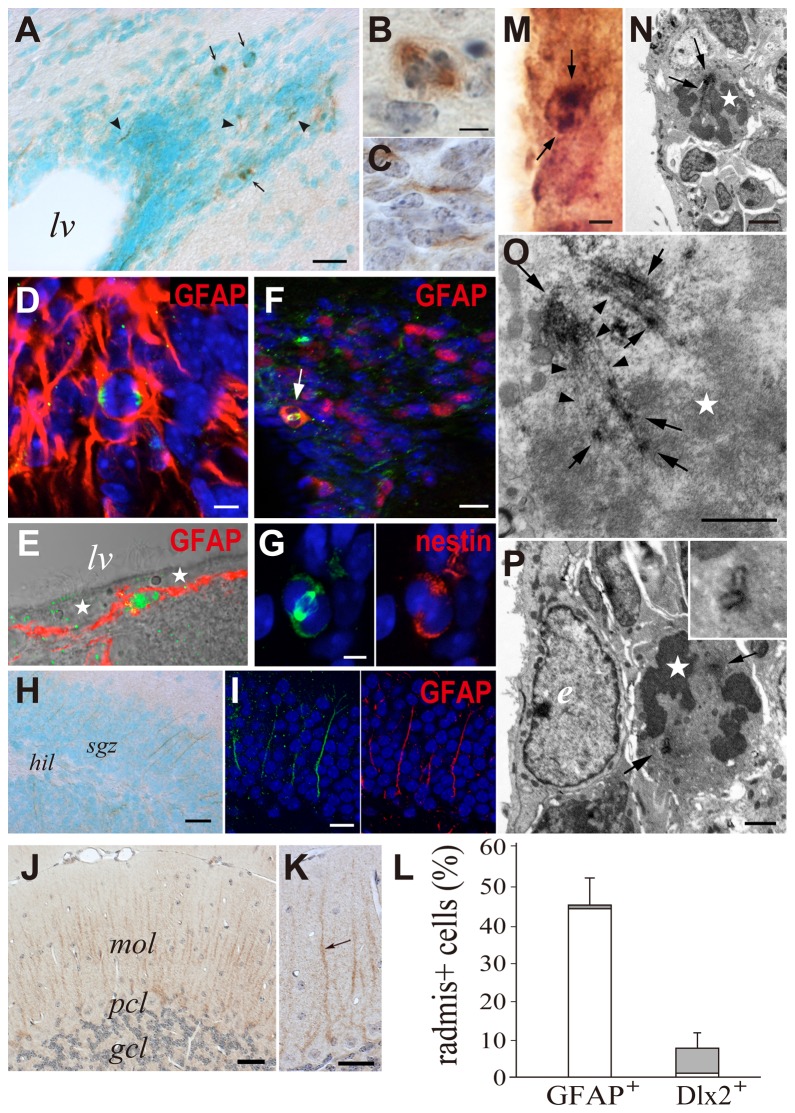
Radmis expression in adult brain. (**A**) SVZ region of the forebrain stained with an anti-radmis antibody. Radmis is expressed in a few SVZ cells (*brown*) surrounding the lateral ventricle. Nuclei are counterstained with methyl green. Radmis immunoreactivity is detected in the mitotic spindles (*arrows*) of SVZ cells, as well as the fine wavy fibers coursing within the SVZ region (*arrowhead*). (**B**, **C**) Magnified view of each radmis-positive SVZ cell, indicating radmis expression in the mitotic spindle of metaphase (**B**), and in short wavy processes (**C**) extending in the SVZ. Nuclei are counterstained with hematoxylin. (**D**) Dorsolateral corner region of the SVZ stained for GFAP (*red*) and radmis (*green*), showing radmis expression in the mitotic spindle of dividing GFAP-positive type-B cells. (**E**) Lateral wall of the lateral ventricle stained for GFAP (*red*) and radmis (*green*). Radmis is expressed in the GFAP-positive astrocyte underneath ciliated ependymal cells (*asterisks*). (**F**) Double-immunostaining of the SVZ for radmis (*green*) and Dlx2 (*red*). *Arrow* indicates a radmis- and Dlx2-positive cell. (**G**) Magnified view of the dividing radmis-positive SVZ cell that simultaneously expresses nestin. (**H**) Dentate gyrus of the hippocampus. Low level expression of radmis is observed at the cellular processes coursing through the SGZ of the dentate gyrus. (**I**) Magnified view of the developing dentate gyrus double-immunostained with radmis (*green*) and GFAP (*red*). (**J**) Cerebellum. Radmis is detected in the radially aligned fibers of Bergman glia, albeit at a much lower level. (**K**) High-power view of Bergman glial fibers (*arrow*). (**L**) Quantification of radmis-positive type-B and type-C cells in SVZ region. The number of radmis^+^ GFAP^+^ cells or radmis^+^ Dlx2^+^ cells were counted in the SVZ region (n = 3 male mice at 8 weeks in age), and the ratio of radmis^+^ GFAP^+^ cells / total GFAP^+^ cells, or radmis^+^ Dlx2^+^cells / total Dlx2^+^ cells were calculated. *Solid* *bars* represent the percentage of the radmis^+^ and phosphoH3^+^ mitotic cells. *Open* *bars* represent the percentage of the radmis^+^ but phosphoH3^-^ cells. Data were expressed as the mean ± SEM (%). (**M**–**P**) Ultrastructural localization of radmis in mitotic cells in the adult SVZ. (**M**) Optical microscopic image of the SVZ region stained with an anti-radmis antibody. A single neural progenitor cell immunopositive for radmis was visualized by a DAB reaction followed by osmification. *Arrows* show the radmis-positive mitotic spindle. (**N**) Electron micrograph of the same section re-embedded and analyzed by EM. Radmis immunoreactivity was detected at the spindle (*arrows*) of a mitotic progenitor cell, which had condensing chromosomes (*asterisk*), and contacted with the lateral ventricle with the cytoplasmic protrusion. (**O**) Higher magnification view of (N), showing the radmis distribution on spindle microtubules (*arrowheads*). Extensive immunoreactivity of radmis was also detected between each fine microtubule extending parallel to each other (*arrows*). (**P**) Electron micrograph of another mitotic cell showing radmis expression in spindle poles (*arrows*). Inset shows a magnified view of the centrosomes, indicating radmis immunoreactivity on the centrioles. An adjacent ependymal cell that had an electron lucent nucleus (*e*) was immunonegative for radmis. Scale bars: *A*, *H*; 25 μm; *B*–*E*, 5 μm; *F*, 20 μm; *G*, 2.5 μm; *M–N*-, 2 μm; *O–PM–N*, 1 μm; *I*, 10 μm; *J*, 50 μm; *K*, 10 μm. *lv*, lateral ventricle; *mol*, molecular layer; *pcl*, Purkinje cell layer; *gcl*, granular cell layer; *sgz*, subgranular zone; *hil*, hillus.

Immunohistochemical analysis was performed to examine the spatio-temporal pattern of radmis expression in postnatal brain. Although the expression of radmis was dramatically reduced in most parts of the CNS during early postnatal development, immunohistochemical analysis indicated that the expression of radmis persisted in the CNS during postnatal development, albeit at a lower level than that observed in the embryonic CNS. At P6, radmis-positive cells were found in the wall of the lateral ventricles, and dorsolateral corner of the SVZ bordered by the striatum and overlying the corpus callosum (Figure 4A). This region is known to be proliferative and contains not only glial precursors for astrocytes and oligodendrocytes [7,19,22], but also neuronal precursors for the interneurons of the olfactory bulb [29]. Among the large number of cells within the SVZ, radmis was expressed in a few cells bearing processes (Figure 4C, *arrowhead*), and cells undergoing mitosis (Figure 4C, *arrows*). These radmis-positive SVZ cells also expressed nestin (Figure 4F, G). Interestingly, radmis expression was frequently associated with the numerous thick processes extending from the ventricular surface into the SVZ, as shown in Figure 5B. Double immunostaining revealed that these cells bearing processes corresponded to the immature ependymal cells or postnatal radial glia that were positive for nestin (Figure 4H). Morphologically, these radmis-positive cells appeared to resemble embryonic radial glia (Figure 3I). Expression of radmis in postnatal radial glia/immature ependymal cells was also observed in several distinct CNS regions. In the SVZ surrounding the 4^th^ ventricle of the pontine, radmis was expressed in nestin-positive cells with longitudinally aligned long processes (Figure 4D, E). However, such expression of radmis was only observed during early postnatal development. In the adult brain, radmis expression was not detected in ependymal cells (Figure 5). Therefore, it is possible that radmis expression occurs in neural progenitors with the morphological or functional properties of radial glia, including the postnatal radial glial fraction. This is consistent with a previous electron microscopy study that showed that the early postnatal VZ (P7) is composed of the immature ependymal cells, in addition to the radial glial cell bodies that remain proliferative, display interkinetic nuclear migration and serve as progenitors of new neurons [30].

A dynamic pattern of radmis expression was also observed in the developing cerebellum. During early postnatal development, the external granule cell layer (EGL) covers the surface of the developing cerebellum, and is composed exclusively of proliferating neuronal progenitors. At P6, the expression of radmis was detected in the EGL (Figure 4I). As neurogenesis progresses, two populations of cells appear in the EGL: cells proliferating in the upper portion of the EGL (EGLa), and cells undergoing the initial step of neuronal differentiation deeper in the EGL (EGLb) [22,31,32]. The expression of radmis was observed in mitotic cells within the EGLa (Figure 4K, *arrow*), but not in NeuN-positive postmitotic granule neurons within the EGLb (Figure 4L). These data indicate that radmis expression is primarily associated with CNS regions where mitotic activities of NSPCs persist. We also noticed that radmis was expressed in the radially aligned fibers of Bergmann glial cells, which were nestin-positive, and coursing from the inner Purkinje cell layer through the EGL to the pial surface (Figure 4K, *yellow*).

In the hippocampus, pyramidal neurons in the CA regions are formed before birth, whereas the majority of the granule cells in dentate gyrus are generated in the early postnatal period. The hippocampal NSPCs were reorganized to establish the secondary proliferative zone, subgranular zone (SGZ), in the dentate gyrus [33] This SGZ maintains throughout adulthood and continues to generate granule neurons. We examined the radmis expression at P7 hippocampus. As shown in Figure 4M, radmis-positive cells were sparsely distributed in the developing hippocampus. In the dentate gyrus, radmis was detected in the progenitors that had the radially aligned process in the granular layer (Figure 4M, *inset*) and occasionally showed the mitotic figure (Figure 4N, *arrows*). Double immunostaining showed that these radmis expressing cells were nestin-positive NSPCs (Figure 4N).

### Radmis expression in the adult CNS

Consistent with the immunoblot analysis, radmis-positive cells were rarely detected in adult brain (Figure 1D). However, closer microscopic inspection revealed a small number of radmis-positive cells residing in the SVZ region surrounding the lateral ventricles (Figure 5A). Immunoreactivity for radmis was observed in the mitotic spindles of dividing cells (arrows in Figure 5A, B) and their cellular processes (arrowheads in Figure 5A, C). These thick and wavy radmis-positive processes were frequently observed in the dorsolateral corner of the SVZ region surrounding the lateral ventricles, and appeared to extend within the SVZ (Figure 5A, C). On the other hand, radmis immunoreactivity was not detected in other SVZ areas such as the SVZ region surrounding the 3^rd^ and 4^th^ ventricles, or the Sylvius aqueduct (data not shown). Two major phenotypes of proliferative progenitors have been described in the adult SVZ, based on expression of the intermediate filament protein GFAP [34]. Type-B cells express GFAP (astrocyte-like) and give rise to rapidly dividing transit-amplifying type-C cells that are not immunopositive for GFAP. Type-B cells are relatively quiescent and may be a population of NSCs residing in the adult SVZ, while type-C cells are positive for nestin and Dlx2, and are the direct precursors of neurons that migrate to the olfactory bulb [34]. Double immunostaining demonstrated that radmis was expressed in the mitotic spindles of GFAP-positive dividing NSCs residing in the SVZ (Figure 5D, E). A previous study showed that type-B cells are localized adjacent to ependymal cells, where they extend processes to form a lamina covering for the ependymal layer, or localize at the interface with the striatal parenchyma in adult forebrain [35]. A fate mapping study using transgenic mice further reported that dividing GFAP-positive progenitors have a predominantly bipolar or unipolar morphology that is markedly different from that of the non-dividing multipolar appearance of stellate astrocytes [6]. GFAP- and radmis-double positive cells were observed in the SVZ underneath the ependymal layer with a bipolar morphology (Figure 5E), thereby supporting the expression of radmis in type-B cells. Nevertheless, it was evident that there was a population of radmis-expressing dividing SVZ cells that were devoid of GFAP immunoreactivity (data not shown). These GFAP-negative cells often had a spherical cell body with fewer processes, and showed immunoreactivity for Dlx2 (Figure 5F) and nestin (Figure 5G) in their dividing cytoplasm, indicating the morphological and immunological characteristics of type-C cells [34]. To determine the proportion of type-B and type-C cells that express radmis, we counted the number of radmis^+^ GFAP^+^ cells and radmis^+^ Dlx2^+^ cells in SVZ region. Figure 5L showed that radmis was expressed in the significant population of GFAP-positive type-B cells (46 ± 7.0 %). Triple staining with radmis, phosphoH3 and GFAP revealed that radmis was not only expressed in the type-B cells in mitotic phase, but also expressed in a significant population of type-B cells that are not actively dividing (Figure 5L, *open bar*). Indeed, many type-B cells in interphase showed the radmis immunoreactivity in their cellular processes as shown in Figure 5A, C and E. On the other hand, radmis was detected in a small fraction of Dlx2-positive type-C cells (7 ± 4.3 %). Virtually all of these radmis-positive type-C cells were in mitosis (Figure 5L, *solid bar*), suggesting the rapid elimination of radmis protein in the interphase type-C cells. Taken together, we concluded that radmis expression persists in the NSCs (type-B) and lineage-restricted neuronal progenitor cells (type-C) in adult forebrain.

In addition to the adult SVZ, new neurons are generated in the hippocampal SGZ of adult mammals. In the adult hippocampus, faint immunoreactivity of radmis was observed in radially aligned cellular processes within the granule cell layer (GCL) (Figure 5H), although the level of immunoreactivity was much lower than that observed at the radial processes within the SVZ. Double immunolabeling indicated that these radmis-positive radial processes were also positive for GFAP (Figure 5I). A previous study showed that GFAP-positive radial astrocytes in the SGZ function as the progenitors (also known as type I progenitors) of these new neurons *via* immature intermediate D cells (type II progenitors) that in turn give rise to new postmitotic granule neurons [36]. Radial astrocytes have cell bodies that line the SGZ and the hilar side of the GCL of the dentate gyrus, as well as possess a major radial process that penetrates the GCL [37]. Based on these observations, it was likely that radmis was expressed in the cellular processes of radial astrocytes. We also observed the radmis expression in the mitotic spindles of the type II progenitors in SGZ (data not shown). Interestingly, such radmis expression in specialized astrocytes with radial processes was detected in the adult cerebellum. As shown in Figure 5J, weak immunoreactivity for radmis was observed in the radially aligned Bergmann glial fibers that course from the Purkinje cell layer through the molecular layer to the pial surface in the adult cerebellum (Figure 5K).

### Radmis is a putative microtubule-associated protein (MAP) expressed in NSPCs

To confirm that radmis associates with the microtubules of mitotic spindles in NSPCs, we performed electron microscopy (EM) analysis of the adult SVZ. The electron micrograph in Figure 5N depicts the ultrastructural localization of radmis in a mitotic NSPC that is presumably an NSC (type-B astrocyte). In contrast to the morphological and topological features of NSCs [35], the radmis-positive cell was in contact with the lateral ventricle (Figure 5N), and adjacent to or underneath ependymal cells (Figure 5P). Radmis immunoreactivity was detected on the radially aligned bundle of fine microtubules that formed mitotic polar and kinetochores spindles (Figure 5O). In addition, radmis protein was localized to the walls of paired centrioles (Figure 5P *, inset*), which are composed of nine triplets of microtubules, and crossed perpendicularly to each other in a centrosome microtubule organizing center. It should be also noted that there was considerable immunoreactivity speckled in a narrow space between extending microtubules (Figure 5O *, arrows*). Together with the distribution of radmis immunoreactivity along entire microtubules ranging from the spindle pole to the junction of chromosomes (Figure 5O), we concluded that radmis is a MAP, serving as the fundamental component of the mitotic spindle apparatus.

### Dynamic turnover of radmis during the cell cycle

**Figure 6:**
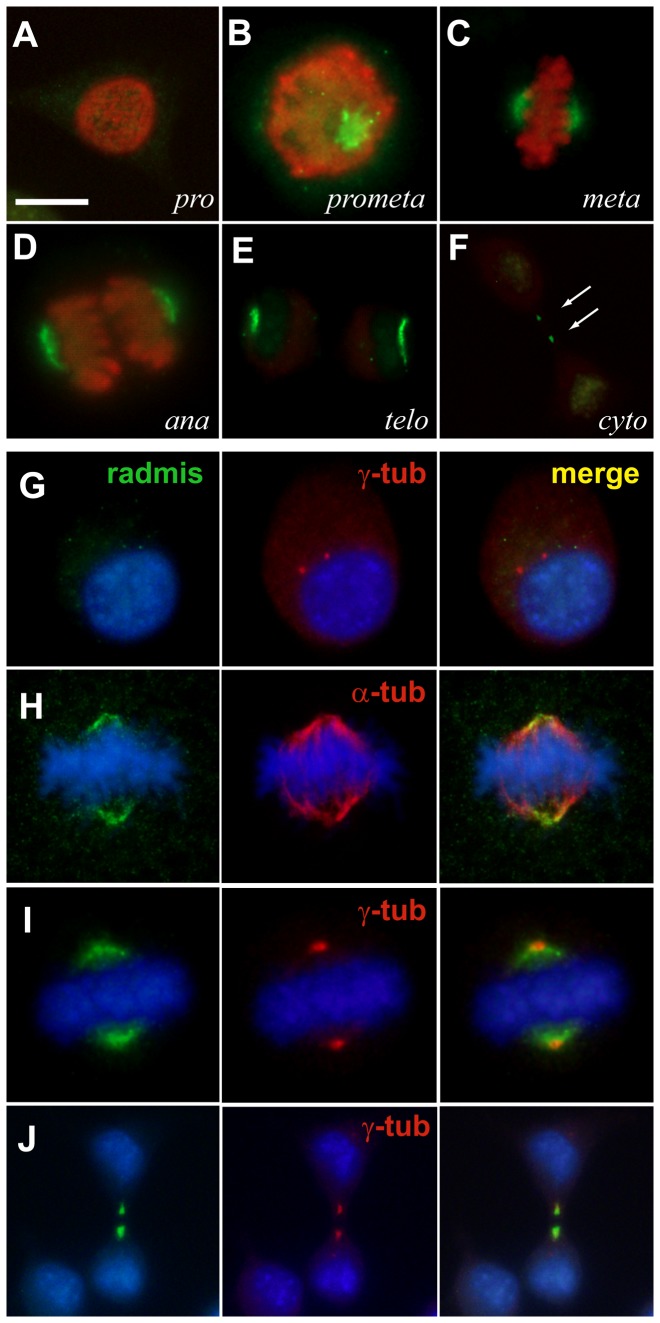
Distribution of radmis during the cell cycle. (**A**–**F**), N2a cells at various stages of mitosis were immunostained for radmis (*green*) and phosphoH3 (*red*) to label M phase chromosomes. (**A**) Prophase. (**B**) Prometaphase. (**C**) Metaphase. (**D**) Anaphase. (**E**) Telophase and (**F**) a cell in cytokinesis. *Arrows* indicate radmis-positive spots of midbodies in the intercellular bridge. (**G**–**J**) Cells in each phase of the cell cycle were stained for radmis (*green*) and α-tubulin or γ-tubulin (*red*). Hoechst was used to identify cell cycle phases (*blue*). (**G**) Interphase cell showing no expression of radmis. (**H**) Metaphase cell showing an overlapped distribution of radmis with α-tubulin on spindle microtubules. (**I**) Metaphase cell, showing radmis localization on γ-tubulin-positive centrosomes. Note that radmis is not expressed on centrosomes in interphase cells. (**J**) Cell undergoing cytokinesis showing colocalization of radmis with γ-tubulin in midbody microtubules. Scale bar: 10 μm.

Our immunohistochemical study indicated that radmis expression was mainly confined to the microtubules of spindles during mitosis, and was absent in the cytoplasmic meshwork of microtubules during interphase of the cell cycle of neural progenitor cells, suggesting cell cycle-dependent regulation of radmis expression. Additionally, we examined several cancer cell lines, including HEK293 and N2a, which showed radmis expression in their mitotic spindle structures. Thus, we next determined the localization of endogenous radmis during the cell cycle of HEK293 cells (Figure 6). The endogenous radmis protein was not detected during G1, S (data not shown) or G2 phases after separation of duplicated γ-tubulin-positive centrosomes (Figure 6G). No radmis localization was observed in the entire microtubule network in the cytoplasm of these interphase cells. From prometaphase to telophase (Figure 6B–E), radmis was localized to mitotic spindle poles (Figure 6I) and spindle microtubules that were positive for α-tubulin (Figure 6H). Later, in accordance with rapid collapse of the mitotic spindles, radmis protein disappeared from spindle microtubules (Figure 6F). During cytokinesis, radmis colocalized with γ-tubulin in midbody microtubules within the intercellular bridge (Figure 6F, J). The M phase specific expression of radmis protein and its association with mitotic spindles suggested the involvement of radmis in the organization of mitotic spindles required for the cell cycle progression during mitosis. The rapid turnover of radmis protein during mitotic exit also implied that its expression might be strictly controlled by a post-translational modification such as rapid protein degradation by ubiquitination.

### *In vivo* overexpression of radmis increases the mitotic rate of cells in VZ/SVZ

**Figure 7:**
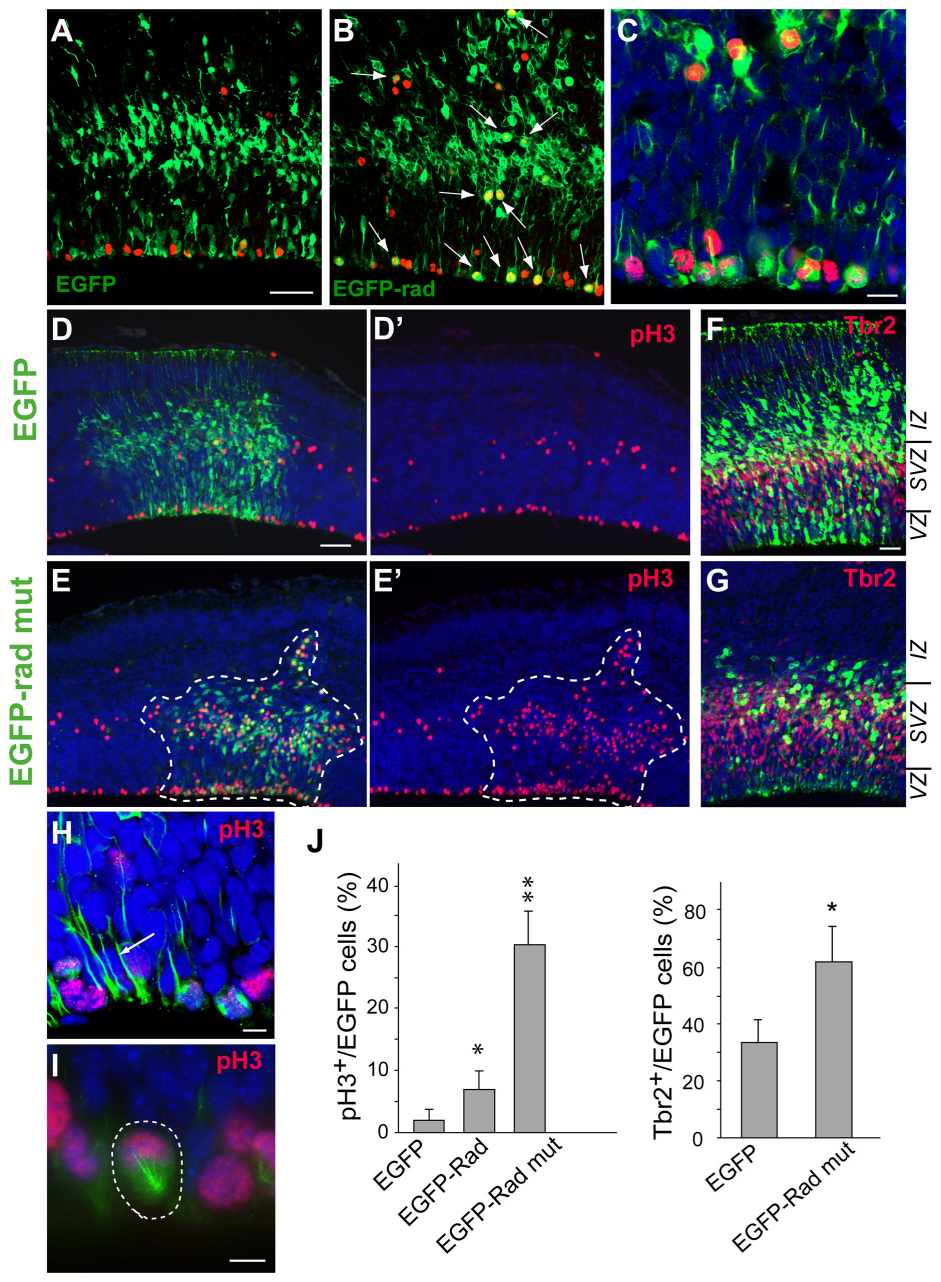
In vivo overexpression of radmis increases the rate of mitotic cells in VZ/SVZ. *In* *utero* electroporation of EGFP-radmis, EGFP-radmis KEN mut (radmis-mut), or control EGFP was performed at E14.5, followed by analysis at 24 h post-electroporation (E15.5). (**A**–**C**) Distribution of cells electroporated with EGFP-radmis (**B**), or control EGFP (**A**) in E15.5 neocortex. Sections were stained for EGFP (*green*) and phosphoH3 (*red*). *Arrows* indicate EGFP and phosphoH3 double-positive cells in the VZ/SVZ. The ventricular surface is at the bottom, and the pial surface is at the top. (**C**) Magnified view of the ventricular surface of an EGFP-radmis electroporated brain. (**D**–**G**) Distribution of cells electroporated with control EGFP (**D**, **F**) or EGFP-radmis-mut (**E**, **G**). (**D**, **E**) Double immunostaining for EGFP (*green*) and phosphoH3 (*red*). Hatched areas in panels E and E’ denote the distribution of cells electroporated with EGFP-radmis-mut. NSPCs overexpressing radmis-mut remain mostly in the SVZ/VZ, and many of them express phosphoH3. (**F**, **G**) Confocal images of electroporated sections stained for EGFP and Tbr2 (*red*). Many cells expressing control EGFP are translocated to the upper IZ toward the pial surface, whereas cells expressing EGFP-radmis-mut remain in the SVZ/VZ and most of them show immunoreactivity for Tbr2. (**H**, **I**) Representative magnified images of radial glia cells that overexpressed EGFP-radmis-mut. EGFP-radmis-mut expression is frequently found in radial fibers (*arrow* in H) and mitotic spindles (*arrowhead*) of radial glial cells, and induces the formation of a monopolar mitotic spindle during mitosis (hatched circle in I) in the VZ. EGFP-radmis-mut (*green*), phosphoH3 (*red*), and DNA (*blue*). (**J**) Quantification of electroporated NSPCs that are positive for phosphoH3 or Tbr2. The ratio of phosphoH3-positive M phase cells, or Tbr2-positive cells, to total EGFP-positive cells in the neocortex was calculated for each electroporation construct, and is presented as the mean ± SEM (%) (group, number of embryo analyzed; EGFP, n = 15; EGFP-Rad, n = 4; EGFP-Rad mut, n = 12). Student’s *t*-tests; * < 0.01, and **p < 0.001 vs. control EGFP. Scale bars: *A*–*B*, 50 μm; *C*, 20 μm; *D*–*E*, 50 μm; *F*–*G*, 20 μm; H, I, 10 μm. *IZ*, intermediate zone; *VZ*, ventricular zone*; SVZ*, subventricular zone.

To understand the functional role of radmis in neurogenesis, we performed the overexpression of *radmis* gene *in vivo*. EGFP-tagged full-length radmis or control EGFP was electroporated into NSPCs in the developing dorsal neocortex at E14.5 during extensive neurogenesis. After 24 h at E15.5, electroporated embryos were harvested and then analyzed. We extensively analyzed embryos electroporated with the radmis transgene. However, radmis transgene expression was hardly observed in most electroporated embryos; out of 60 embryos analyzed, only four embryos expressed the EGFP-radmis transgene. This low frequency of transgene expression did not appear to be due to a failure of gene transfer into cortical cells because we verified simultaneous expression of a red fluorescence protein (DsRed-Express) reporter plasmid that was co-electroporated with the EGFP-radmis plasmid into the same embryos (data not shown). This observation could be explained by the tight (post-translational) regulation of the radmis level *in vivo*, which prevents inappropriate expression of the radmis protein. Nonetheless, we analyzed the embryos that exhibited considerable expression of the EGFP-radmis gene by immunostaining with an anti-phophoH3 antibody, and observed an increased number of phophoH3-positive cells among VZ/SVZ cells (Figure 7A, B). Quantitative double immunostaining analysis with anti-phophoH3 antibodies revealed that approximately 8.0% of cells electroporated with EGFP-radmis also expressed phophoH3, whereas only 1.3% of cells expressed phophoH3 in cortices electroporated with control EGFP (Figure 7J). The majority of EGFP-radmis/phophoH3-positive cells were found in the ventricular surface, which had vertically extending radial processes (Figure 7B, C), indicating their characteristic as radial glia. 

### A stable mutant of radmis protein enhances the accumulation of NSPCs in the mitotic phase

**Figure 8:**
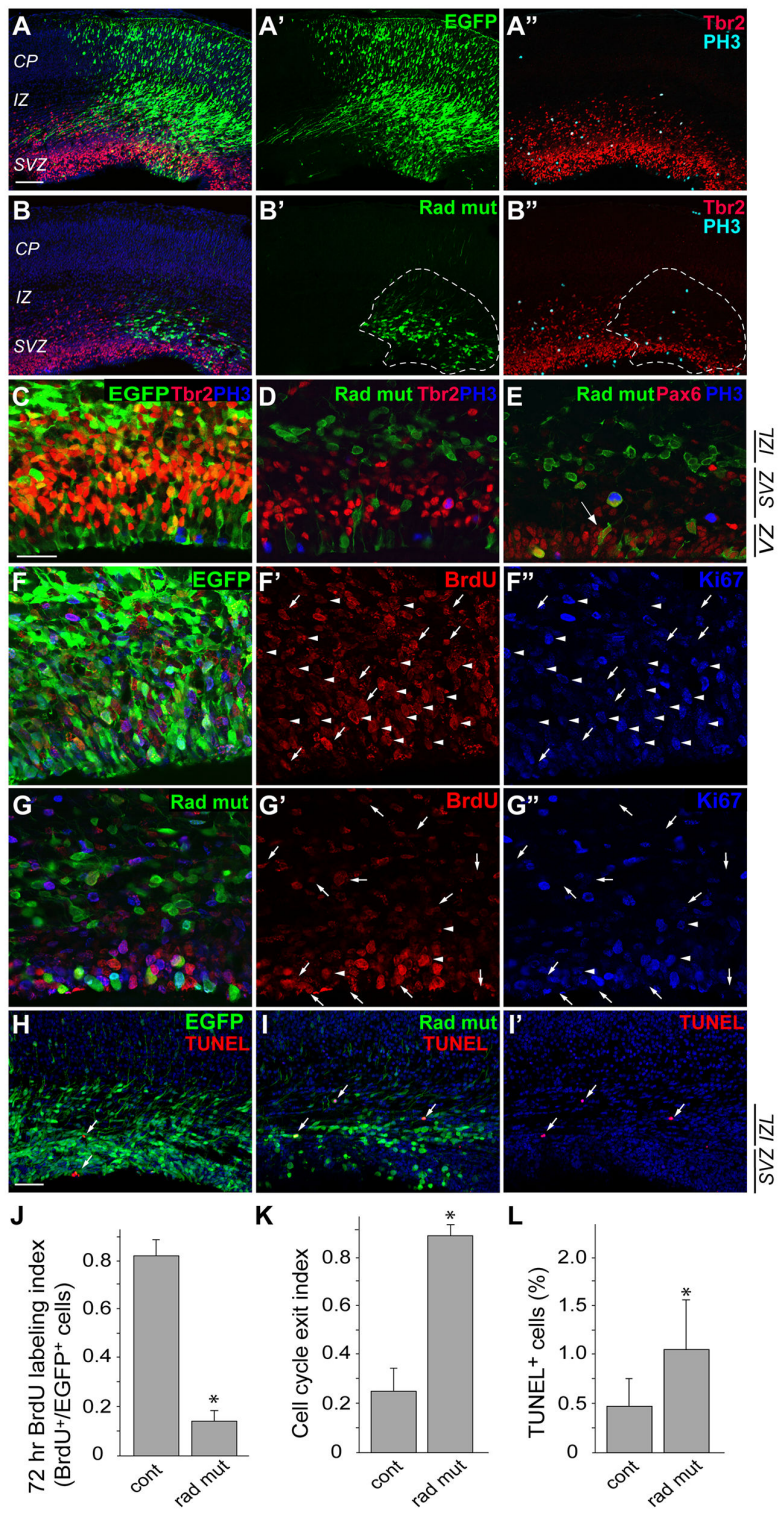
Sustained expression of radmis results in decreased cell proliferation and increased cell-cycle exit of NSPCs. Control EGFP or EGFP-radmis-mut constructs were electroporated into E14.5 embryonic brains that were analyzed after 72 h (E17.5). (**A**, **B**) Confocal images of E17.5 brain sections electroporated with control EGFP (**A**) or EGFP-radmis-mut (**B**). Sections through the neocortex were stained for EGFP (*green*), Tbr2 (*red*), phosphoH3 (*light blue*), and DNA (*blue*). Hatched areas in panels B and B’ show the cell distributions of cells electroporated with EGFP-radmis-mut. By E17.5, the Tbr2-positive SVZ layer became much thinner upon electroporation of EGFP-radmis-mut (compare with the thickness of the neighboring Tbr2-positive SVZ outside the hatched areas in panel B”). (**C**, **D**) Magnified views of VZ/SVZ regions in A and B, respectively. Virtually none of the cells overexpressing EGFP-radmis-mut are Tbr2-positive. (**E**) Confocal image of electroporated brain immunostained for EGFP-radmis-mut (*green*), Pax6 (*red*), and phosphoH3 (*blue*). Compared with the control, the majority of cells overexpressing EGFP-radmis-mut no longer express Tbr2, and most of them translocated to the IZL. *Arrow* indicates persistent expression of radmis-mut in Pax6-positive radial glial cells in the VZ. (**F**–**I**) Prolonged expression of radmis-mut results in reduced cell proliferation, and increased cell-cycle exit and cell death. E14.5 brains were electroporated with control EGFP (**F**) or EGFP-radmis-mut (**G**) constructs. BrdU was consecutively administered into dams after 24 h post-electroporation (E15.5–E17.5), followed by analysis of brains at 72 h (E17.5). Left column shows merged confocal images of sections stained for EGFP (*green*), BrdU (*red*), and Ki67 (*blue*). Middle and right columns show BrdU-, and Ki67-labeled cells, respectively. *Arrowheads* indicate EGFP, BrdU, and Ki67 triple-positive cells, and *arrows* indicate EGFP- and BrdU-positive, but Ki67-negative, cells. (**H**, **I**) Apoptotic cells (*arrows*) were detected by TUNEL staining in control (**H**) or radmis-mut electroporated brains (**I**, **I**’). EGFP (*green*) and TUNEL-positive cells (*red*). (**J**) Quantification of 72 h BrdU labeling index. Data are presented as mean ± SEM (%). Student’s *t*-tests (n = 5 embryos); *p < 0.001. (**K**) Quantification of the cellcycle exit index. The cell cycle exit index was calculated as follows; [(number of EGFP^+^, and BrdU^+^ cells) – (number of EGFP^+^, BrdU^+^, and Ki67^+^ cells)] /(total number of EGFP^+^, and BrdU^+^ cells), and presented as the mean ± SEM (%). Student’s *t*-tests (n = 5 embryos); *p < 0.001. (**L**) Quantification of apoptotic cells. The ratio of TUNEL-positive cells to total EGFP-positive cells was calculated, and is presented as the mean ± SEM (%). Student’s *t*-tests (n = 6 embryos); *p < 0.01. Scale bars: *A*–*B*, 100 μm; *C*–*G*, 50 μm; *H–I*; 50 μm. *IZL*, lower intermediate zone; *VZ*, ventricular zone*; SVZ*, subventricular zone.

The unexpectedly lower frequency of radmis transgene expression among electroporated embryos led us to speculate that the radmis gene product might be very unstable, and the expression level may be tightly controlled *in vivo* by post-translational regulation such as ubiquitin-mediated proteasome proteolysis. Indeed, sequence analysis of the radmis protein revealed the existence of a KEN box motif (Lys-Glu-Asn) in the amino-terminal half (Figure 1A, *asterisks*). The KEN box is a known target sequence of APC/C-Cdh1 [38,39]. It has been reported that the anaphase-promoting complex/cyclosome (APC/C), an E3 ubiquitin ligase, mediates rapid degradation of target substrates including multiple mitotic regulators such as cyclins and cyclin kinases [40]. Cdh1, an activator of APC/C, directly binds to and maintains the activity of APC/C from late anaphase to early G1 phase, mediating proper degradation of target substrates and controlling the exit from the mitotic phase [41]. The APC/C-Cdh1 complex recognizes KEN box-containing proteins as substrates [39]. Substantial database analysis using BLAST revealed that the KEN box motif found in radmis was conserved across species including mouse, rat, chick, dog, cow, and human (data not shown). It also should be noted that TMAP/ckap2 contains a KEN box motif near the N-terminus, and APC/C-Cdh1 mediates KEN box-dependent degradation of TMAP/ckap2 during mitotic exit [42,43]. Thus, we hypothesized that radmis protein might be a target molecule for APC/C-Cdh1, and rapidly degraded *via* the KEN box during mitosis in a similar manner to that of TMAP/ckap2. To test this hypothesis, we constructed a KEN box mutant of full-length radmis protein (EGFP-radmis-mut), in which the KEN (Lys-Glu-Asn) was replaced with AAA (Ala-Ala-Ala), and evaluated its expression *in vivo* using *in utero* gene transfer. Consequently, a large number of embryos (43 out of 45 embryos electroporated) that showed a detectable level of the radmis-mut transgene were collected at a greatly improved frequency, indicating that the KEN box mutant form of radmis served as a non-degradable variant. We analyzed the distribution of EGFP-positive cells in various zones of the developing cortex. As shown in Figure 7D, control EGFP-expressing cells were widely distributed along the cortical axis from the VZ to the intermediate zone (IZ), and cortical plate (CP) at 24 h post-electroporation (E15.5). At this time, forced expression of radmis-mut resulted in substantial alteration in the cell distribution among cortical zones, in which the majority of cells remained in the VZ and SVZ (Figure 7E). Accordingly, fewer cells migrated into the IZ or CP (group, n; control, n = 15; EGFP-radmis-mut, n = 12). Double immunostaining with phophoH3 revealed that a prominent fraction of these accumulated cells within the VZ/SVZ were undergoing mitosis following radmis-mut expression, compared with that of the control (30.0% of electroporated cells were phophoH3-positive) (Figure 7J). Most of these cells in the SVZ also expressed Tbr2 [28], a marker of basal progenitor cells in the developing SVZ (Figure 7F, G, J). The majority of radmis-expressing cells were preserved as the fraction of Tbr2-positive basal progenitor cells in the SVZ, indicating that persistent expression of radmis maintained neural progenitors in a mitotic state. In addition, cells expressing radmis-mut in the VZ exhibited longitudinally aligned processes, the morphological features of radial glia (Figure 7H), and these cells frequently formed the abnormal monopolar spindle during mitosis (Figure 7I). To determine the cell fate of progenitor cells that overexpressed radmis, we carried out *in utero* electroporation at E14.5 and harvested brains at 72 h later (E17.5). In controls, robust expression of EGFP persisted in almost all cells in the distinct layers of the neocortex (Figure 8A). At this time, expression of radmis-mut was almost cleared in the SVZ (Figure 8B), and largely confined to the densely packed cells in the lower region of the IZ (IZL), which overlays the SVZ (Figure 8D, E). Radmis-mut expression was detected in a small fraction of cells in other regions of the neocortex including the VZ, the upper region of the IZ and CP. Radmis-mut-expressing cells in the IZL were devoid of Tbr2 expression, and had round- to oval-shaped cell bodies with fine and wavy processes (Figure 8D), which are indicative of non-dividing cells that have just migrated out of the SVZ. Intriguingly, double immunostaining with Tbr2 revealed a significant reduction in the number of Tbr2-positive basal progenitors and shrinkage of the SVZ in the area where the radmis-mut construct was introduced (area surrounded by the dotted line in Figure 8B). The number of Tbr2-positive cells in the area where the radmis-mut gene was electroporated was quite low compared with that of Tbr2-positive cells in the neighboring ipsilateral area of the cortices, where gene transfer was not achieved (53 ± 34 Tbr2^+^ cells/2000 cortical cells in the radmis-mut electroporated area, n = 9 *vs* 283 ± 148, n = 7, in the non-electroporated area). It also should be noted that expression of radmis-mut was persistently detected in a small number of Pax6-positive radial glia in the VZ [28], which had radial processes (Figure 8E). 

### Prolonged radmis expression inhibits NSPCs to re-enter the cell cycle

**Figure 9:**
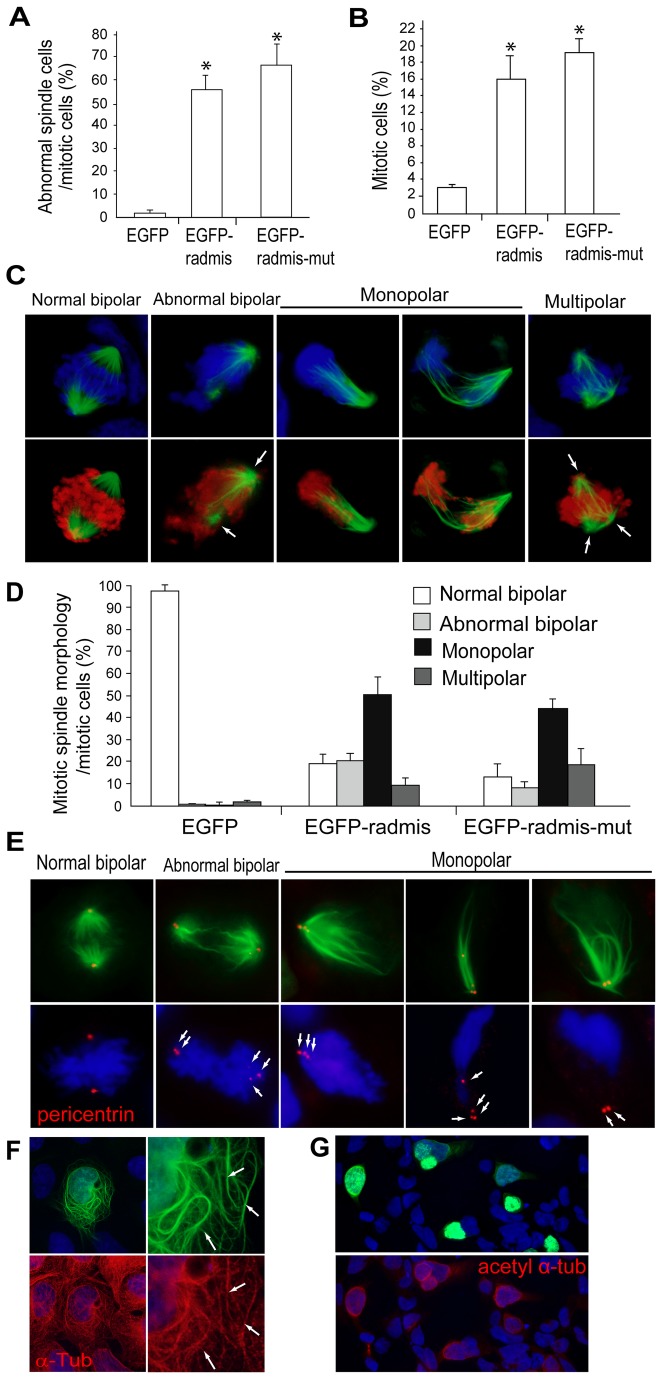
Radmis gain-of-function induces mitotic arrest accompanied by a mitotic spindle defect. HEK293 cells were transfected with an EGFP-radmis, EGFP-radmis-mut, or EGFP control plasmid. At 44 h post-transfection, cells were fixed for analysis. (**A**) Quantification of cells exhibiting abnormal spindles among EGFP- and phosphoH3-positive mitotic cells. The morphology of mitotic spindles was analyzed by immunostaining for α-tubulin (data not shown). (**B**) Quantification of the mitotic index. The percentages of phosphoH3-positive mitotic cells among EGFP-, EGFP-radmis-, or EGFP-radmis-mut-transfected cells were calculated. (**A**, **B**) Data are obtained from four independent transfection experiments and presented as means ± SEM (%), and *p < 0.01 for EGFP control (Student’s t-test). EGFP-radmis (n = 428 cells), EGFP-radmis-mut (n = 370 cells), and control EGFP (n = 535 cells). . (**C**) Representative images of mitotic cells with aberrant spindles. Mitotic spindles observed in EGFP-radmis-transfected cells were categorized into normal bipolar, abnormal bipolar, monopolar, and multipolar. PhosphoH3 (*red*) and DNA (*blue*). An abnormal bipolar spindle is characterized by disorganized interpolar microtubules (*arrows*), and a multipolar spindle is characterized by extra spindle pole(s) (*arrows*). (**D**) Quantification of each type of mitotic spindle among EGFP-, EGFP-radmis-, or EGFP-radmis-mut-transfected cells. Data are obtained from six independent transfection experiments and presented as means ± SEM (%). EGFP (n = 97 mitotic cells), EGFP-radmis (n = 163 mitotic cells), EGFP-radmis-mut (n = 213 mitotic cells). (**E**) Centrosome labeled with an anti-pericentrin antibody (*red*) after radmis gain-of-function. Monopolar spindles in radmis-overexpressing cells were frequently accompanied by unseparated centrosomes. *Arrows* indicate centrosomes. DNA (*blue*). (**F**) Overexpression of EGFP-radmis induces microtubule bundling at interphase. Cytoplasmic microtubules in an interphase cell overexpressing EGFP-radmis (*green*) were visualized by α-tubulin staining (*red*). Right panels are higher magnifications showing radmis deposition in abnormally thick and winding or stiff microtubule bundles in the cytoplasm (*arrows*). (**G**) Cells transfected with EGFP-radmis (*green*) were immunostained for acetylated α-tubulin (*red*). Note that untransfected cells exhibit only a negligible level of acetylation of α-tubulin.

A shrunken and exhausted SVZ might reflect the outcome of accelerated differentiation or inhibition of re-entering the cell cycle of Tbr2-positive progenitor cells. To determine whether this SVZ shrinkage coincided with changes in the proliferative activity of radmis-mut-expressing cells, we administrated BrdU into electroporated pregnant dams from E15.5 (24 h post-electroporation) to E17.5, followed by collection of brains. We then determined the 72 h BrdU labeling index within the EGFP-positive cell population. Immunostained sections revealed that the majority of cells expressing radmis-mut did not incorporate BrdU between 24–72 h post-electroporation (Figure 8F, G). As shown in Figure 9J, only a small fraction (13%) of cells among radmis-mut-expressing cells had the ability to proliferate, whereas approximately 80% of cells could re-enter the cell cycle in controls (n = 5, comparison between littermates), indicating that fewer cells re-entered S phase following radmis-mut expression. We also performed immunostaining for Ki-67 on the same sections to detect actively proliferating cells, and evaluated the cell cycle exit index (Figure 8F, G). Figure 8K shows that radmis-mut expression led to a striking increase in the number of cells exiting the cell cycle, which was up to 3.6-fold, compared with that in the controls (n = 5). This elevated cell cycle exit index was comparable with the decrease in the cell proliferation rate. Which cell-fate will the radmis-mut expressing progenitors follow? To determine whether the accelerated cell cycle exit causes increased neuronal or glial differentiation, we immunostained electroporated E17.5 brains for a post-mitotic neuronal marker, NeuN, and βIII-tubulin, or glial markers S100β and GFAP. However, we failed to obtain evidence that these cells correspond to post-mitotic neurons or glia, since they were immunonegative for these cell-type markers (data not shown).We next evaluated the apoptotic cells in the electroporated brains by TUNEL staining, because the aberrant daughter cells frequently die from apoptosis. As shown in Figure 9H and I, some fractions of the radmis-mut expressing cells underwent cell death in the IZL, whereas the electroporation of control EGFP resulted in significantly fewer dead cells (Figure 8L).

Taken together, these data demonstrated that persistent expression of radmis-mut directs a reduction in the proliferative activity of progenitor cells and a concomitant increase in cell cycle exit, after which their progenies frequently follows the aberrant differentiation or cell death. It is likely that the defect in the ability of cycling SVZ cells to re-enter the mitotic phase results in the exhausted basal progenitor population and shrinkage of the SVZ over several cell cycles. The transient increase in the number of progenitor cells observed at 24 h post-electroporation (E15.5), which were positive for phophoH3 and Tbr2, might represent an accumulation of abnormal basal progenitor cells in the SVZ, which are in a state of mitotic arrest or cell cycle delay. Many of the progenies leaving the cell cycle may have defects in their ability to survive or migrate toward the CP, resulting in the cell death or accumulation of aberrant cells in the IZL, which exhibit atypical characteristics different from those of post-mitotic immature neurons or glia.

### Radmis functions in formation of mitotic spindles, and its overexpression arrests mitosis

To explore the cellular mechanism by which the dysregulated radmis expression causes the perturbation of NSPCs mitosis, we further analyzed the effect of ectopically expressed EGFP-radmis or EGFP-radmis-mut on the cultured HEK293 cells. At M phase, radmis overexpression dramatically induced severe defects in mitotic spindle formation in HEK293 cells. The mitotic index (the percentage of mitotic cells) was determined by counting the percentage of phosphoH3-positive cells among EGFP-expressing cells at 44 h after transient transfection. We found that EGFP-radmis-, EGFP-radmis-mut-, and control EGFP-expressing cells exhibited a mitotic index of 16.0% (n = 428 cells), 19.3% (n = 370 cells), and 3.1% (n = 535 cells), respectively, suggesting that the balance of M phase entry and exit was significantly disrupted (Figure 9A, B). Morphological analysis of individual cells indicated that a large number of mitotic cells exhibited spindle defects upon overexpression of the EGFP-radmis or the EGFP-radmis-mut, in which 25-28 fold increase in the percentage of cells showed abnormal mitotic spindles, and these cells constituted approximately 58% and 66% of all mitotic cells, respectively (Figure 9A). The spindle phenotype caused by the radmis expression was independent of the wild-type protein or the KEN-box mutant, and the most prevalent spindle defect commonly observed in radmis overexpressing cells was the formation of monopolar mitotic spindles containing highly bundled and elongated microtubules (Figure 9C). The majority of EGFP-radmis (52%) or EGFP-radmis-mut-expressing mitotic cells (45%) showed a monopolar spindle, whereas no such cells were observed among EGFP control cells (Figure 9D). These abnormal spindles frequently extended several long and thick branches (monopolar in Figure 9C), displaying striking differences from the stereotypical normal bipolar spindles (normal bipolar in Figure 9C). In accordance with the abnormal monopolar spindle formation, the condensed chromatids were arranged in a disorganized manner and failed to line up on an equatorial metaphase plane (Figure 9C). The absence of a definitive metaphase plane also implied that these cells were arrested in a prometaphase-like state and had not yet progressed into normal anaphase. In addition to monopolar spindle formation, we frequently observed abnormal bipolar spindles or multipolar spindles upon overexpression of radmis (21% and 10% of mitotic cells, respectively), or radmis-mut (8% and 18% of mitotic cells, respectively), which is characterized by disorganized and irregularly extended interpolar microtubules (Figure 9C). These observations suggested that the spindle function was severely impaired in cells overexpressed radmis. To examine whether the formation of monopolar spindles is due to centrosomal abnormalities, we labeled the centrosome with an antibody against pericentrin, a conserved integral component of the filamentous matrix of the centrosome [44]. At M phase, cells overexpressed EGFP-radmis often showed either two centrosomes at the pole of the monopolar spindle, or abnormally amplified centrosomes at the vicinity of the spindle pole (Figure 9E, *arrows*). However, even though cells had multiple centrosomes clustered within a monopolar spindle, these centrosomes appeared to be replicated (Figure 9E), suggesting that the formation of monopolar spindles was not due to a failure in centrosome duplication prior to metaphase. Rather, it is likely that a defect in centrosome separation during M phase progression resulted in the monopolar spindle formation of radmis-overexpressing cells.

At interphase, radmis gain-of-function induced microtubule polymerization that formed abnormally thick microtubule bundles in the cytoplasm (Figure 9F). Colocalization with α-tubulin confirmed that this fibrillar structure was highly bundled microtubules. Closer examination of microtubules showed a thick bundle-like pattern that is characteristic of hyper-polymerization and -stabilization (Figure 9F, *arrows*). Indeed, the induction of hyper-stabilization of microtubules was demonstrated by immunostaining with an antibody against acetylated α-tubulin, which detects stabilized microtubules [45]. It was evident that radmis was colocalized with acetylated α-tubulin in thick bundles of microtubules in the cytoplasm, whereas the acetylation of α-tubulin was not induced in untransfected cells (Figure 9G). Such hyper-stabilization of microtubules was also observed in N2a cells transfected with EGFP-radmis (data not shown). Considering these findings, it was possible that the abnormal monopolar spindle formation during cell division was attributable to segregation failure of duplicated microtubules spindles that had accumulated the overexpressed radmis protein and exhibited the hyper-stabilized property. Through the same or similar mechanism, the ectopically expressed radmis may cause the monopolar spindles in NSPCs *in vivo*, as observed in Figure 7I. The impaired spindle assembly might induce the mitotic arrest or cell cycle delay of NSPCs, leading the substantial accumulation of abnormal NSPCs in SVZ.

### Radmis loss of function disrupts mitotic spindles and chromosome segregation

**Figure 10:**
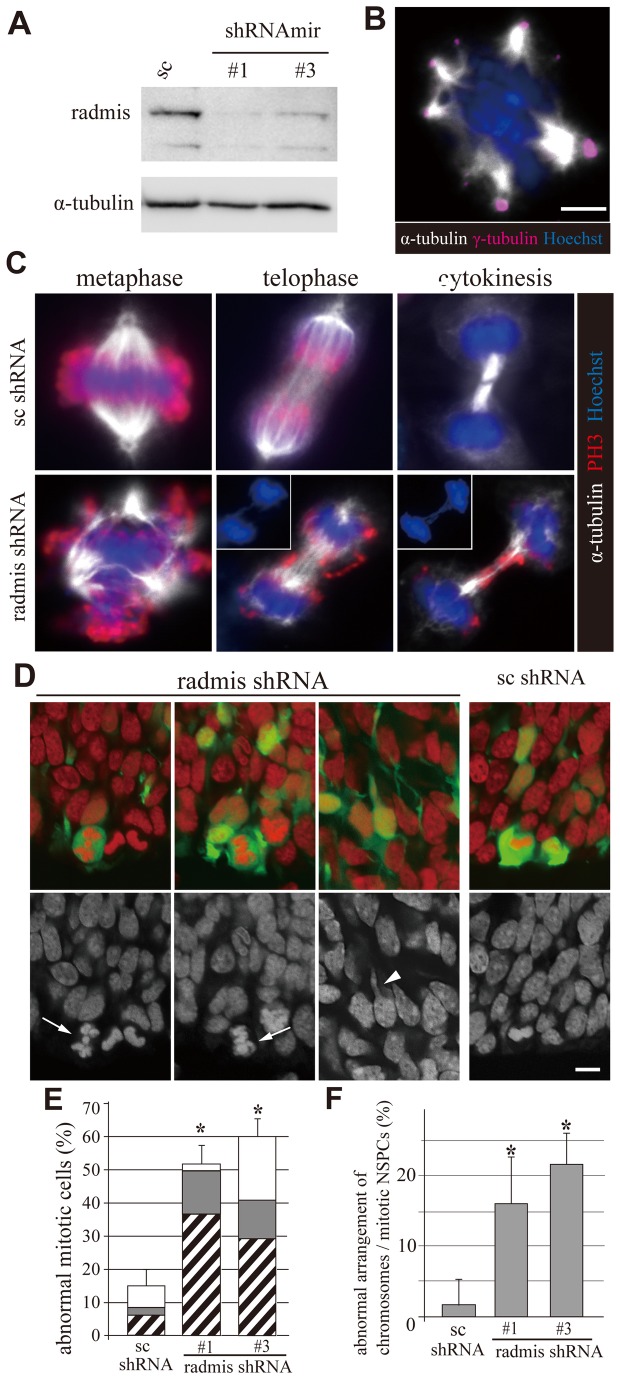
Depletion of radmis induced the multipolar spindles and the catastrophe of chromosome segregation. (**A**-**C**, **E**) NIH3T3-13C7 cells were treated with EGFP-radmis shRNAmir (shRNA#1 and #3), or scrambled shRNA (control). (**A**) Immunoblot analysis demonstrating depletion of endogenous radmis at 48 h post-transfection. Total cell extracts were probed with the radmis antibody. Antibody to α-tubulin was used to demonstrate equal loading. (**B**) Representative image of a multipolar spindle induced by the radmis depletion. Centrosomes were stained with γ-tubulin (*magenta*), and microtubules were stained with α-tubulin (*white*). Hoechst DNA staining (*blue*). (**C**) Representative images of mitotic cells, showing the failure of chromosome segregation. *Upper* *panels*; control cells transfected with scrambled shRNA. Lower panels; cells transfected with radmis shRNA. *Left* *panel*; abnormal multiple chromosome configuration. Chromosomes were distorted and moved away from metaphase plate at metaphase, along with the multipolar spindle and irregular distribution of phosphoH3. *Right* *two* *panels*; abnormal chromosome bridge with the ectopically emerged phosphoH3 in telophase and cytokinesis. Insets; DNA staining, denoting the ectopically extending chromatid between daughter nuclei. α-tubulin (*white*), phosphoH3 (*red*), and DNA staining (*blue*). (**D**) Radmis loss of function *in* *vivo*. EGFP-tagged shRNAmir constructs were electroporated *in* *utero* into the NSPCs at E13.5, and analyzed 24 h later. Representative images of radmis depleted cells. *Arrows* indicate the abnormal chromosome organization in mitotic NSPCs. *Arrowhead* shows the neuron migrating in intermediate zone, which has a distorted nuclear protrusion toward the cortical surface. Electroporated cells with shRNA (*green*), and DNA staining with PI (*red*). (**E**) Quantification of mitotic cells exhibiting abnormal chromosome arrangement among radmis depleted NIH3T3-13C7 cells. Data are obtained from four independent transfection experiments and presented as means ± SEM (%) (group, mean ± SEM (%), cell number analyzed; shRNA#1, 52.0 ± 4.9*, n = 1321; shRNA#3, 60.4 ± 5.4*, n = 1561; scrambled shRNA, 15.0 ± 5.3, n = 1544).Unpaired *t*-test; *p < 0.01 *vs* scrambled shRNA. *Hatched* *bar*, cells showing the abnormally branched chromosomes with multipolar spindle at metaphase; *closed* *bar*, cells showing chromosome bridge at telophase and cytokinesis; *open* *bar*, cells in cytokinesis exhibiting the unequal size and different morphology between two daughter nuclei. (**F**) Quantification of mitotic cells with the abnormal organization of chromosomes among the shRNA-treated NSPCs undergoing cell division in embryonic VZ/SVZ. Data are presented as the mean ± SEM (%) (group, mean ± SEM (%), animal number analyzed; shRNA#1, 16.4 ± 6.5*, n = 9; shRNA#3, 21.5 ± 4.5*, n = 10; scrambled shRNA, 2.0 ± 4.0, n = 7).Unpaired *t*-test; *p < 0.01 *vs* scrambled shRNA. Scale bars: *B*-*C*, 10 μm; *D*, 10 μm.

To assess the effect of radmis loss-of-function, we screened and generated the microRNA-adapted short hairpin shRNA (shRNAmir) constructs, which has been shown to greatly increase gene silencing efficiency [29]. We identified two different shRNAmir constructs (shRNA1, and shRNA3) capable of knocking down the expression of endogenous radmis in cultured NIH3T3/13C7 cells (Figure 10A). NIH3T3/13C7 was selected for the knockdown analysis because of the moderate expression level of endogenous radmis, and the suitability for the observation of individual mitotic chromosome. Western blot analysis indicated that transfection with shRNA1 and shRNA3 induced the reduction of 90% and > 95% of radmis protein, respectively, compared to scrambled shRNA. Cells transfected with scrambled shRNA did not affect the morphology of mitotic spindles through metaphase into cytokinesis (Figure 10C). In contrast, radmis knockdown induced a severe disorganization of the mitotic spindles and chromosome separation. The most prevailing phenotype of radmis knockdown was a failure of chromosome alignment on the equatorial plane (metaphase plate) at metaphase, accompanied by the formation of multipolar spindles. More than 50% of radmis depleted cells exhibited the alignment failure of chromosomes in metaphase (group, mean ± SEM (%); shRNA#1, 52.0 ± 4.9*; shRNA#3, 60.4 ± 5.4*; scrambled shRNA, 15.0 ± 5.3; unpaired *t*-test; *p < 0.01).These chromosomes did not line up at the metaphase plate, and several abnormal-shaped chromosomes appeared to be hanging out of the metaphase plate. Staining with α- and γ-tubulin indicated that this irregular configuration of chromosomes was accompanied by the abnormal multipolar spindles extending from the multiple γ-tubulin-positive centrosomes (Figure 10B). Compared to the normal spindle microtubules, each mitotic spindle in radmis depleted cells appeared significantly smaller because the spindle pole radiated shorter microtubule branches (Figure 10B, C). Such shrinkage of spindle microtubules seemed to be the opposite phenotype to the hyper- stabilized microtubules induced by the overexpression of radmis (Figure 9). No monopolar spindle was observed in radmis depleted cells. 

Another striking phenotype induced by radmis depletion was the chromosome bridge, which is an abnormal chromatin that bridges the two separating daughter nuclei in anaphase. As shown in Figure 10C, many of the radmis depleted cells formed the long fine chromosome bridge, which was visible with DNA dye in telophase and even in cytokinesis stage. In these radmis depleted cells, we concomitantly detected the ectopic distribution of phosphoH3 outside the daughter nuclei, even where DNA staining was invisible (Figure 10C), suggesting a catastrophe of chromosome segregation and a failure of the symmetrical nuclear division. Indeed, radmis depleted cells frequently generated two daughter cells which had an unequal size and different nuclear morphology during cytokinesis (Figure 10E).

Next, we tested the impact of radmis loss of function on NSPCs *in vivo*. EGFP-tagged shRNAmir constructs (shRNA #1 and #3) driven by CAG promoter were electroporated *in utero* into the NSPCs lining the ventricular wall of telencephalon at E13.5, and harvested embryos 24 h later. Electroporation with the scrambled shRNA did not disturb the alignment of chromosomes in metaphase plate, or the nuclear morphology of NSPCs (Figure 10 D). In addition, the progression of mitosis appeared normal. By contrast, knockdown of radmis resulted in a severe disarrangement of chromosomes at metaphase in NSPCs (group, n, mean ± SEM (%); shRNA#1, n = 9, 16.4 ± 6.5*; shRNA#3, n = 10, 21.5 ± 4.5*; scrambled shRNA, n = 7, 2.0 ± 4.0; Unpaired *t*-test; *p < 0.01). In most cases, chromosomes in knockdown NSPCs were often twisted and aberrantly protruded from the metaphase plate (Figure 10D *arrows*). We also observed the distorted morphology of nucleus having a bulged extension toward the cortical surface in a small number of neurons migrating in the intermediate zone (Figure 10D). We hardly detected such abnormal nuclei among embryos electroporated with the control shRNA (Figure 10F). These results indicated that radmis loss of function disrupts the mitotic spindles and induces the failure of chromosome segregation *in vitro* and *in vivo*. A strictly regulated level of radmis during cell-cycle would be crucial for the bipolar spindle formation and chromosome segregation for the proper proliferation of NSPCs.

## Discussion

### Radmis is expressed in NSPCs throughout development

Our immunostaining observations using an affinity-purified polyclonal antibody against radmis revealed that radmis is preferentially expressed by embryonic and adult NSPCs *in vivo*, as well as cultured NSPCs. In addition to mitotic spindles, radmis is localized in the radially aligned long processes of NSPCs, neuroepithelial cells in the early embryonic stage and radial glia (apical progenitor cells) in the VZ during the mid-late embryonic period. A previous study has reported that radial glia differentiate into ependymal cells at the perinatal period. Consistently, during postnatal development, radmis expression is found in differentiating nestin-positive ependymal cells that possess a radial fiber reaching into the parenchyma through the SVZ. Such radmis expression in undifferentiated ependymal cells might indicate a competence of these cells to generate neural progenies as postnatal radial glia. Consistent with this idea, a previous study [7] suggested that neonatal radial glial-like cells not only serve as progenitors for many neurons and glia soon after birth, but also give rise to adult SVZ stem cells. In the adult brain, radmis expression is retained by a small number of NSPCs including GFAP-positive type-B cells in the SVZ. Interestingly, radmis appears to be consistently expressed in a discrete cell population, namely radial glial-like cells that have the common morphological feature of radial projection [46]. In the adult cerebellum, we showed radmis expression in the radial processes of Bergmann glia. Bergmann glia are important for precise arborization of Purkinje cells, but little is known about other roles of this cell population after cerebellar development. Recent studies [47-49] have shown that Bergmann glia possess stem cell characteristics *in vitro*, and also express the same key markers (Sox1/Sox2/Sox9) as those of NSPCs, suggesting that these cells correspond to the recently reported stem cells in the adult cerebellum. Although the significance of radmis expression in Bergmann glia remains unclear, it is possible that radmis expression reflects the characteristics of adult NSPCs that have so far only been identified in the SVZ of lateral ventricles and the subgranular region of the hippocampus.

### Radmis as a novel regulator of microtubule dynamics during neural development

Radmis expression is dynamically regulated during NSPC division. EM revealed that radmis is uniformly distributed along each microtubule bundle of spindles in addition to centrioles during mitosis, whereas its expression promptly diminishes at interphase. Thus, it is highly likely that radmis is a novel MAP in NSPCs, which is transiently expressed in the mitotic apparatus including mitotic spindles and centrioles. Radmis overexpression *in vitro* caused the formation of an aberrant monopolar spindle at the mitotic phase, and hyper-stabilized cytoplasmic bundles consisting of acetylated α-tubulin-positive microtubules at interphase, suggesting a role of radmis in polymerization/stabilization of microtubules. By contrast, radmis knockdown induced the abnormal multipolar spindles emanating from the multiple centromeres, frequently accompanied by the failure of chromosome alignment at the metaphase plate. Each microtubule branch of the multipolar spindle appeared to be shorter in length, indicative of the destabilization of spindle microtubules. This effect of radmis depletion sharply contrasts with the phenotypes induced by radmis overexpression; the monopolar spindle formation and stabilization of microtubules. Microtubule stabilizing property of radmis appears to be consistent with the general property of MAPs. Several MAPs affect bipolarity by controlling microtubule stability. For example, MAP4 overexpression results in formation of monopolar spindles [50]. A recent study further demonstrated that MAP4 is essential for maintaining spindle position and the correct cell division axis in cells [51]. Likewise, radmis may regulate the precise positioning of microtubules which is required to establish the correct cell division plane of NSPCs. Basically, the number of times that NSPCs re-enter the cell cycle determines the size of the brain, and strictly controlled timing of cell division is crucial to determine the total number of neurons that each NSPC can generate during neurogenesis [9]. During brain development, rapid and drastic morphological rearrangements of microtubules occur at various stages to control the cell division of NSPCs and migration of new neurons. The dynamic balance between polymerization and depolymerization of mitotic spindle microtubules is also required for dividing NSPCs to drive the process of chromosome segregation during mitosis [11]. The spindle can also segregate many cell fate-determining factors such as mPar3 and numb [52,53]. Recently, much attention has been focused on the relationship between MAPs and neurogenesis by NSPCs [11,54]. In particular, studies of genes responsible for developmental brain disorders in humans have provided evidences that the proteins interacting with the mitotic spindle play vital roles in normal neurogenesis progression. For example, the mutation in abnormal spindle-like microcephaly-associated protein (ASPM) are the most common cause of primary microcephaly characterized by a small brain and mental retardation in humans. ASPM is a microtubule minus end-associated protein at the spindle poles during mitosis, and is involved in spindle organization and orientation to regulate the frequency of symmetric *vs* asymmetric NSPC division [13,54]. Another MAP involved in neurogenesis is doublecortin-like kinase (DCLK). DCLK shares high homology with doublecortin (DCX). DCLK, but not DCX, is highly expressed in regions of active neurogenesis in the neocortex, and regulates the formation of bipolar mitotic spindles during cell division and the progression of M phase of NSPCs [12]. Considering that many NSPCs fail to re-enter the cell cycle after *in vivo* ectopic expression of a radmis mutant, it is conceivable that radmis might play a role in regulation of spindle formation that is essential for the maintenance of dividing NSPCs during neurogenesis. It would be interesting to examine whether *radmis* is a candidate gene associated with developmental brain disorders in humans.

### Possible molecular machinery relevant to radmis

We further indicated the possibility that radmis is unstable and rapidly degraded at mitotic exit. Based on the sequence, we speculated that APC/C-Cdh1 mediates ubiquitination of radmis protein via the KEN box motif. The ubiquitin E3 ligase complex APC/C is essential for coordination of cell cycle transitions, including mitotic exit by promoting degradation of important key regulators of the cell cycle such as aurora kinase, securin, and Plk1 [40]. APC/C is conserved among species and its substrate specificity depends on two adaptor proteins, Cdc20 and Cdh1, and the KEN box is frequently found in the substrate proteins of APC/C-Cdh1 [39]. Using *in utero* electroporation, we showed that the introduction of a mutant KEN box leads to drastically enhanced protein stability *in vivo*, supporting the idea that radmis is a novel substrate of APC/C-Cdh1 during mitosis. These observations imply that the tightly regulated protein level of radmis is crucial for normal assembly of the mitotic spindle and progression of the cell cycle or cell cycle exit in brain development. Consistent with this idea, a previous genetic study of *cdh1* indicated that APC/C-Cdh1 is required to prevent unscheduled proliferation of Sox2/Sox9-positive NSPCs in the adult brain [55]. In addition, our knockdown experiment indicated the disorganization of chromosome alignment at metaphase, and the chromosome bridge formation in cytokinesis. These abnormalities could be caused by the cohesion defect of sister chromatids [33]. APC/C is also known to trigger the events leading to proteolysis of Cohesin [56], allowing the sister chromatids to separate at anaphase. Thus, it is possible that radmis is involved in the molecular cascade through APC/C to Cohesin to operate the separation machinery of sister chromatids.

Radmis shows a certain similarity (16% amino acid identity) with TMAP/ckap2 that was originally identified in humans as a gene highly expressed in tumors including hematopoietic tumors [57] and gastric carcinomas [58]. As shown in Figure 1A, TMAP/ckap2 also contains a KEN box motif, and co-localizes with microtubules. Recent *in vitro* studies [42,43] indicated that TMAP/ckap2 is a substrate of APC/C-Cdh1, and degraded via the KEN box during mitotic exit, indicating that tight regulation of the TMAP/ckap2 protein level is critical for normal mitotic progression. In addition, ectopic expression of a non-degradable KEN box mutant of TMAP/ckap2 leads to mitotic arrest with monopolar spindles containing bundled microtubules in Hela and HEK293 cells [42,43]. Based on these comparable molecular properties during cell division, we propose that *radmis* and *TMAP/ckap2* genes constitute a novel MAP family with microtubule-stabilizing properties in mammals. Nonetheless, it remains unknown whether TMAP/ckap2 shares a similar expression pattern with that of radmis in the nervous system. Double-knockout experiment with *radmis* and *TMAP/ckap2* genes may unequivocally reveal the function of this MAP family in neurogenic mitosis.
